# Fluorescently Labelled ATP Analogues for Direct Monitoring of Ubiquitin Activation

**DOI:** 10.1002/chem.202001091

**Published:** 2020-04-28

**Authors:** Daniel Hammler, Katrin Stuber, Fabian Offensperger, Martin Scheffner, Andreas Zumbusch, Andreas Marx

**Affiliations:** ^1^ Department of Chemistry University of Konstanz Universitätsstraße 10 78457 Konstanz Germany; ^2^ Department of Chemistry and Center for Applied Photonics University of Konstanz Universitätsstraße 10 78457 Konstanz Germany; ^3^ Department of Biology University of Konstanz Universitätsstraße 10 78457 Konstanz Germany

**Keywords:** ATP, fluorescent probes, PET, UBA1, ubiquitin

## Abstract

Simple and robust assays to monitor enzymatic ATP cleavage with high efficiency in real‐time are scarce. To address this shortcoming, we developed fluorescently labelled adenosine tri‐, tetra‐ and pentaphosphate analogues of ATP. The novel ATP analogues bear — in contrast to earlier reports — only a single acridone‐based dye at the terminal phosphate group. The dye's fluorescence is quenched by the adenine component of the ATP analogue and is restored upon cleavage of the phosphate chain and dissociation of the dye from the adenosine moiety. Thereby the activity of ATP‐cleaving enzymes can be followed in real‐time. We demonstrate this proficiency for ubiquitin activation by the ubiquitin‐activating enzymes UBA1 and UBA6 which represents the first step in an enzymatic cascade leading to the covalent attachment of ubiquitin to substrate proteins, a process that is highly conserved from yeast to humans. We found that the efficiency to serve as cofactor for UBA1/UBA6 very much depends on the length of the phosphate chain of the ATP analogue: triphosphates are used poorly while pentaphosphates are most efficiently processed. Notably, the novel pentaphosphate‐harbouring ATP analogue supersedes the efficiency of recently reported dual‐dye labelled analogues and thus, is a promising candidate for broad applications.

## Graphical abstract

**Novel fluorescently labelled ATP analogues for direct monitoring of ubiquitin activation**: Fluorescently modified adenosine tri‐, tetra‐ and pentaphosphates (ATP) analogues changing their fluorescence properties upon enzymatic turnover have been synthesised and applied to both E1s found in humans. Pentaphosphates were found to have superior processing and with the used acridone fluorophore the read‐out showed both fluorescence intensity and lifetime, making this assay versatile in application.

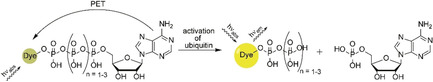

## Introduction

**Figure 1:**
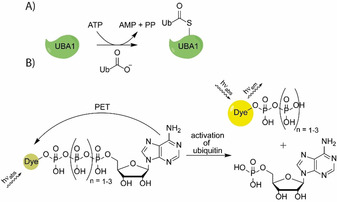
A) Ubiquitin (Ub) activation by E1 (UBA1) with ATP. Adenylated Ub is loaded on UBA1 by the formation of a thioester. B) Concept of mono‐labelled ATP analogues as UBA 1 sensors that are investigated in this study: When in close proximity to the nucleobase adenine, the fluorescent dye is quenched as a result of photoinduced electron transfer (PET). After enzymatic release of the phosphate chain, the fluorescence is restored.

Ubiquitination — the covalent modification of proteins by the 76 amino acid protein ubiquitin (Ub) — is a ubiquitous protein modification with fundamental roles in numerous cellular processes including protein degradation, DNA damage repair, cell cycle regulation and gene expression.^1^, ^2^ Malfunction of the ubiquitination system contributes to a broad variety of human diseases like cancer, diabetes or neurodegenerative disorders.^3^, ^4^ For the attachment of Ub to substrate proteins, the consecutive action of at least three classes of enzymes is needed. In the first step, Ub is activated by a ubiquitin‐activating enzyme (E1) at the consumption of ATP. Thereby Ub is adenylated and then transferred to the active‐site‐ cysteine of the E1 to form a thioester with the C‐terminal glycine carboxylate of Ub^5^ (Figure 1 A). By transthiolation, Ub is transferred to a ubiquitin‐conjugating enzyme (E2). Finally, by the aid of ubiquitin‐protein ligases (E3), Ub is covalently connected to the target protein by forming an isopeptide bond with the *ϵ*‐amino group of a lysine residue.

In humans, two E1s (UBA1 and UBA6) for Ub are known.^6^, ^7^ Quantifying and following E1 activity in real‐time is of great importance to study for example, effectors, but means to do so are sparse. Available E1 activity assays include SDS‐PAGE analysis of E1/E2‐Ub thioester conjugates by Western Blot,^8^ radio‐labelling of the involved proteins with ^125^[I]^9^ or ^32^[P],^10^ FRET between Ub and E1^11^ or enzyme‐coupled spectrophotometric assays for phosphate determination.^12^ Drawbacks of these assays are that they are either laborious, do not allow continuous read‐out or are dependent on additional enzymes of the downstream cascade. To fill this gap, we have recently developed a time‐resolved ATPase sensor (TRASE) based on a Förster resonance energy transfer (FRET) pair embedded within the ATP scaffold to continuously monitor ATP‐dependent enzymes.^13^, ^14^ This ATP FRET probe sensor uses two fluorescent dyes, a donor and an acceptor dye, that are attached to the terminal phosphate group and to adenine, respectively. By employing the TRASE assay, we found that γ‐modified triphosphates are poorly accepted by UBA1 whereas δ‐modified tetraphosphates turned out to be better substrates.^15^

In order to improve the substrate properties of ATP‐based E1 sensors, we synthesized and investigated new ATP analogues that contain only one dye and differ in the length of the phosphate chain for their propensity to visualize E1 activity. Indeed, we identified a fluorescent dye with an acridone core structure that is efficiently quenched by the canonical nucleobase adenine.

While triphosphates were poorly processed, we found that also in this case acceptance is improved by elongation of the phosphate chain, that is, tetraphosphates are better accepted than triphosphates. In fact, elongation of the phosphate chain to pentaphosphate resulted in even better analogues enabling us to follow the activation of Ub by both enzymes, UBA1 and UBA6, in real‐time.

## Results and Discussion

**Figure 2:**
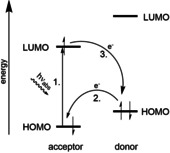
PET and the donor and acceptor frontier orbital energies.

Recently, we developed γ‐modified ATP analogues that are suitable as model compounds for monitoring enzymatic ATP consumption by fluorescence lifetime readout. Compared to other probes, these compounds contain only one instead of two fluorophores, which promises better enzymatic acceptance. Fluorescence lifetime changes between the intact and the cleaved ATP analogues are caused by the quenching of fluorescence by the nucleobase adenine^16^ (Figure 1 B). The quenching is caused by photoinduced electron transfer (PET). In PET, the efficiency for electron transfer rates and thus for quenching can be estimated by using the Rehm‐Weller equation and/or by comparing the involved highest occupied molecular orbital (HOMO) energy levels.^17^, ^18^, ^19^ The PET process starts with excitation of the acceptor chromophore by irradiation (step 1 in Figure 2). This promotes an electron into the lowest unoccupied molecular orbital (LUMO) of the acceptor. In case the HOMO of a neighbouring donor molecule in the immediate vicinity is higher in energy than the HOMO of the acceptor molecule, an electron is transferred from donor to acceptor (step 2) and fluorescence is quenched. The cycle is closed by transfer of an electron from the acceptor LUMO to the donor HOMO. This process can only take place for distances between acceptor and donor on a nanometre scale, that is, in the intact ATP analogue. Enzymatic cleavage of the phosphate chain, however, leads to an immediate separation of the dye‐adenine pair by diffusion such that fluorescence of the dye is restored.

PET‐based quenchers that have previously been employed in biological assays are guanine (−5.33 eV; calculated from its redox potential)^20^ and tryptophan (−4.90 eV; calculated from its redox potential),^21^ both of which possess high HOMO energies.^22^, ^23^, ^24^, ^25^, ^26^ By contrast, due to its lower HOMO energy (−5.78±0.01 eV),^16^ adenine has only rarely been used as quencher. To develop new suitable dye‐adenine pairs, we used photoelectron spectroscopy in air (PESA) to determine HOMO energy levels for hydrophilic dyes. With this approach, we recently were able to investigate different fluorophores like rhodamines and BODIPYs from dry thin films that suited for fluorescence lifetime readout when attached to ATP.^16^ While we could demonstrate enzymatic processing and real‐time detection of these compounds, their main drawback was the relatively short lifetime of these dyes. This made the detection of lifetime changes especially under biologically relevant conditions difficult.

**Scheme 1:**
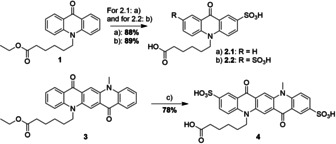
Synthesis of disulfonated acridone (upper panel) and quinacridone (lower panel) derivatives: a) conc. H_2_SO_4_, 120 °C, 20 h. b) 65 % SO_3_ in conc. H_2_SO_4_, rt, 24 h. c) conc. H_2_SO_4_, 110 °C, 20 h.

In order to overcome this shortcoming, we now investigated fluorescent dyes with long fluorescent lifetimes. Acridone and quinacridone as well as their derivatives are interesting candidates for our purpose, especially because they are extremely photostable,^27^ have a long fluorescence lifetime of 14 ns and 22 ns, respectively, and show no spectral pH dependency in the biological relevant range from pH 5–9.^28^ However, only few examples are reported where in particular acridone derivatives under the name Puretime 14 (PT14) were used for fluorescence lifetime‐based biological assays.^29^, ^30^, ^31^, ^32^ For functionalization and attachment of acridone or quinacridone to ATP, we followed a known synthesis strategy. Initially, we created water‐soluble compounds from the organic pigments by introducing sulfonic acid residues (Scheme 1).^28^ We expected that sulfonation would also lower the energy of the frontier orbitals that in turn would result in efficient quenching of the dye when being in close proximity to adenine.

Both *N*‐alkylated fluorescent dyes were treated with hot sulphuric acid, which yielded Sacridone‐COOH (**2.1**) and S_2_quinacridone‐COOH (**4**), respectively. For disulfonation of the functionalized acridone, we used 65 % SO_3_ in H_2_SO_4_ to yield S_2_acridone‐COOH (**2.2**).

**Figure 3:**
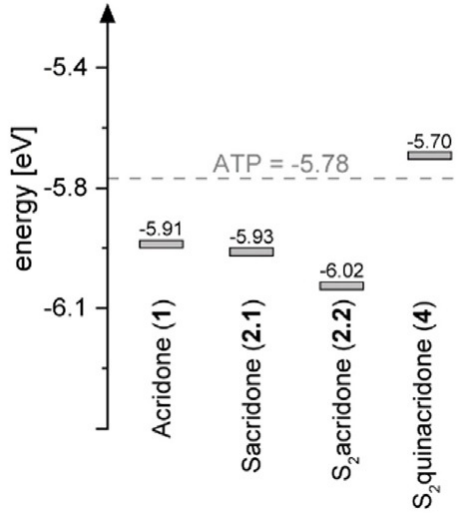
Relative HOMO energies in air. The dashed line represents the value for ATP.

With the water‐soluble compounds **2.1**, **2.2** and **4** in hand, we performed PESA to obtain insights into the HOMO energies in air. To be efficiently quenched by adenine, the HOMO energies of the fluorescent dyes should be lower than that of ATP (−5.78±0.01 eV)^16^ (Figure 3).

**Scheme 2:**
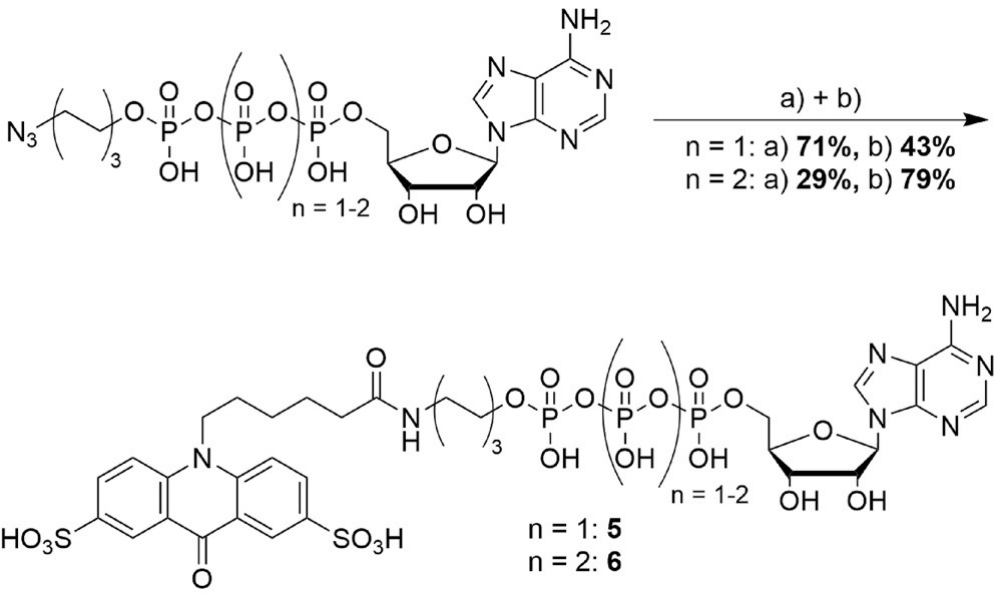
Synthesis of S2acridone functionalized tri‐ and tetraphosphates. a) TECP, MeOH, Et_3_N, H_2_O, 18 h, 4 °C. b) 1. S_2_acridone‐COOH, TSTU, Et_3_N, DMF, rt, 3 h, 2. S_2_acridone‐NHS, 0.1 m NaHCO_3_, rt, 1 h.

We found that all functionalized acridones (**1**, **2.1** and **2.2**) possess lower HOMO energies than ATP, whereas the sulfonated S_2_quinacridone (**4**) is higher in energy. For the acridone derivatives (**1**–**2.2**), we measured HOMO energies of −5.91±0.05 eV (**1**), −5.93±0.03 eV (**2.1**) and −6.03±0.02 eV (**2.2**). These findings nicely confirmed our expectation that the HOMO energies are decreasing, the more electron‐withdrawing groups are introduced. Since the measured HOMO energies are lower than that of ATP, we moved on to the synthesis of a γ‐modified ATP analogue bearing disulfonated acridone. Here, we followed a well‐established synthesis strategy. In brief, commercially available ATP was alkylated,^33^ followed by azide reduction with tris(2‐carboxyethyl)phosphine (TECP) and subsequent NHS ester coupling by activating the carboxylic acid of S_2_acridone‐COOH (**2.2**) with *N*,*N*,*N’,N*’‐tetramethyl‐*O*‐(*N*‐succinimidyl)uronium tetra‐fluoroborate (TSTU) in situ which was finally coupled without purification to the free amine to yield Ap_3_‐Dye (**5**) (Scheme 2).

As a first proof of concept, we determined the fluorescence lifetimes of Ap_3_‐Dye (**5**) in the intact and cleaved state. As a model enzyme for cleavage, we used the ATP hydrolysing phosphodiesterase I from *Crotalus adamanteus* (Snake Venom Phosphodiesterase, SVPD). Before cleavage, we measured a fluorescence lifetime value of 8.71±0.10 ns. By addition of SVPD, the triphosphate is cleaved and the quencher adenine is released as AMP and separated from the fluorophore. Here, we measured a value of 15.64±0.25 ns, corresponding to an absolute lifetime change of 6.93±0.27 ns. With typical experimental errors in fluorescence lifetime determination in the range of 100 ps, this difference is well suited to use lifetimes for quantification of ATP analogue cleavage. Additional absorption and emission spectra recorded before and after cleavage with SVPD did not reveal a spectral shift assuming a low interaction between the sulfonated dye and the nucleobase adenine (data are shown in the Supporting Information).

Encouraged by these results, we next focused on the synthesis of ATP analogues with longer phosphate chains to determine their acceptance by UBA1. From our previous experiments on the enzymatic turnover of ATP analogues by UBA1, we knew that modified δ‐tetraphosphates are superior substrates compared to modified γ‐triphosphates in UBA1‐mediated activation of Ub.^15^ We speculated that extended phosphate anhydride chains in the present ATP analogue will also increase the activity towards UBA1. Therefore, we synthesized a modified δ‐tetraphosphate by activating ATP with 1‐ethyl‐3‐(3‐dimethylaminopropyl) carbodiimide (EDC) to form a trimetaphosphate which was treated with 1‐(6‐azido)hexyl phosphate according to the literature [34]. Afterwards the product was reduced and coupled to S_2_acridone‐NHS (Ap_4_‐Dye (**6**), Scheme 2). Interestingly, for Ap_4_‐Dye (**6**) we found a fluorescence lifetime of 8.74±0.03 ns, which is similar to that of Ap_3_‐Dye (**5**) (8.71±0.10 ns).

**Scheme 3:**
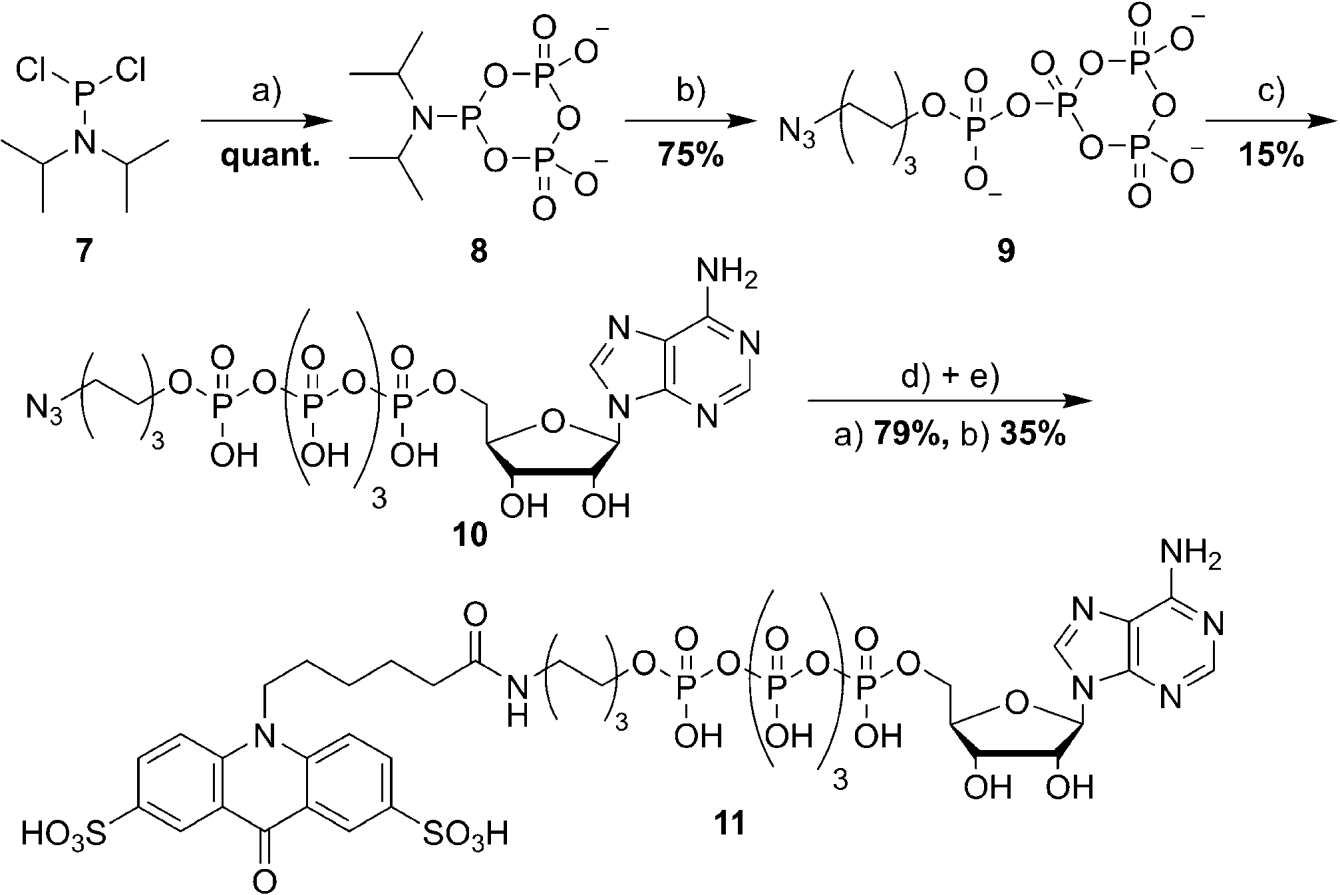
Synthesis Scheme of unsymmetrical pentaphosphates: a) PP*2 Bu_4_N^+^, MeCN, DIPEA, 0 °C, 10 min. b) 1. 6‐azidohexyl phosphate*Bu_4_N^+^ MeCN/DMF, ETT, rt, 3 h. 2. mCPBA, rt, 20 min. c) AMP*Bu_4_N^+^, MeCN/DMF, MgCl_2_, rt, 1.5 h. d) TECP, MeOH, Et_3_N, H_2_O, 18 h, 4 °C. e) S_2_acridone‐NHS, 0.1 m NaHCO_3_, 1 h, rt.

Findings from other groups showed that modified pentaphosphates are even better substrates for nucleic acid polymerases than shorter congeners^35^, ^36^, ^37^ which motivated us to additionally synthesize an *ϵ*‐modified alkylated pentaphosphate. Described synthesis routes of alkylated pentaphosphates that follow P^V^‐N activation and subsequent substitution often lead to unwanted side‐products.^35^, ^38^, ^39^, ^40^ Therefore, we decided to explore a known iterative polyphosphorylation approach strategy.^41^ The reaction is conducted in several steps without purification in one pot. Only modified monophosphates are needed as starting materials, which can be easily obtained in high yields or are even commercially available. Scheme 3 shows an overview of the synthesis of Ap_5_‐Dye. Diisopropylamino dichlorophosphine (**7**) is reacted first with pyrophosphate to form a cyclic pyrophosphoryl P‐amidite (**8**) which is coupled to 6‐azidohexyl phosphate by subsequent oxidation to form 1‐(6‐azido)hexyl phosphoryl cyclotriphosphate (**9**). The cyclic trimetaphosphate is then opened by adding adenosine monophosphate as nucleophile and MgCl_2_ to yield *ϵ*‐*O*‐6‐azidohexyl)‐adenosine‐O5’‐pentaphosphate (**10**) in a one‐pot reaction with an overall yield of 11 %. Reduction of the azide followed by coupling of the activated S_2_acridone using its NHS ester yielded the desired compound (Ap_5_‐Dye (**11**), 28 % over two steps, Scheme 3). For the Ap_5_‐Dye **11**, the fluorescence lifetime of 9.86±0.19 ns is slightly higher than that observed for the tri‐ and tetraphosphates **5** and **6** (8.71±0.10 ns and 8.74±0.03 ns, respectively). The same fluorescence lifetime of the free dye was measured when **10** was treated with SVPD as for (**5**+**6**) (15.64±0.25 ns). The reduced quenching for longer distances between dye and quencher pairs is in accordance to the literature where efficient PET‐quenching takes place on a sub‐nanometre scale.^42^

**Figure 4:**
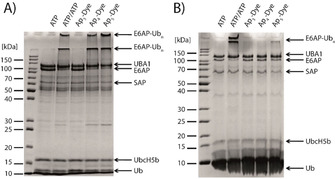
Investigation of the substrate scope of UBA1 (A) and UBA6 (B) for ATP analogues using an E6AP auto‐ubiquitination assay. UBA1 and UBA6 activity is monitored by the appearance of poly‐ubiquitinated E6AP (E6AP‐Ubn) and the simultaneous decrease of the band intensity of free Ub and E6AP in a Coomassie blue stained SDS‐PA gel. All probes were preincubated with rSAP. ATP/Ap_n_‐Dye=150 μm, MgCl2=10 mm, DTT=1.25 mm, UBA1/UBA6=300 nm, UbcH5b=500 nm, E6AP=250 nm and Ub=30 μm, 90 min, 30 °C.

With all three compounds in hand, we tested them towards their performance in an E6AP auto‐ubiquitination assay that was previously shown to be well‐suited to qualitatively evaluate the acceptance of ATP analogues by UBA1 (Figure 4).^15^, ^43^ All reaction mixtures were pre‐treated with recombinant shrimp alkaline phosphatase (rSAP) which dephosphorylates all terminally bound phosphate groups, for example, of ATP to its nucleoside,^44^ while it leaves terminally modified nucleotides unaffected (like for Ap_n_‐Dye). This ensures that the observed activity is due to the Ap_n_‐Dye analogue and does not originate from potential contaminations of natural ATP. After preincubation, rSAP was inactivated by heating the mixture to 65 °C for 5 minutes. In the first lane in Figure 4 A and B, reactions are depicted in which ATP is hydrolysed by rSAP and, thus, auto‐ubiquitination of E6AP is not observed. As positive control, an additional amount of ATP was added after inactivation of rSAP which results in the formation of poly‐ubiquitinated forms of E6AP (E6AP‐Ub_n_) and a concurrent decrease of free Ub and non‐modified E6AP, respectively. The results obtained clearly indicate that Ap_3_‐Dye (**5**) is poorly processed by UBA1, while the extension of the phosphoanhydride chain increases activity. In fact, Ap_5_‐Dye (**11**) was found to be an excellent substrate for UBA1, superior also to Ap_4_‐Dye (**6**).

Similar to UBA1, UBA6 is known to support E6AP autoubiquitination.^7^, ^8^, ^9^, ^10^, ^11^, ^12^, ^13^, ^14^, ^15^, ^16^, ^17^, ^18^, ^19^, ^20^, ^21^, ^22^, ^23^, ^24^, ^25^, ^26^, ^27^, ^28^, ^29^, ^30^, ^31^, ^32^, ^33^, ^34^, ^35^, ^36^, ^37^, ^38^, ^39^, ^40^, ^41^, ^42^, ^43^, ^44^, ^45^ Hence, to determine the ability of UBA6 to employ our ATP analogues, we also followed the formation of polyubiquitinated E6AP. As before, rSAP was used to remove terminally bound phosphates and an additional amount of ATP was added as positive control after heat inactivation of rSAP, which resulted in the formation of polyubiquitinated E6AP (Figure 4 B). Within 90 minutes, formation of E6AP‐Ub_n_ was neither observed with Ap_3_‐Dye (**5**) nor with Ap_4_‐Dye (**6**) whereas the pentaphosphate analogue (**11**) was accepted as substrate. However, the efficiency appeared to be lower than for UBA1.

Next, we investigated whether real‐time experiments are feasible by using UBA1 as well as UBA6 (Figure S2.1 and S2.2). A standard multiwell plate reader was used for this purpose and the increase in intensity at 450 nm of S_2_acridone was followed. The same picture as already seen in the E6AP auto‐ubiquitination assay described above was observed. With Ap_3_‐dye (**5**) no increase in fluorescence intensity and thus no activated Ub was detected whereas the tetraphosphate and the pentaphosphate are processed by UBA1 and UBA6. Again, the pentaphosphate **11** shows its superior properties over Ap_4_‐Dye (**6**). With UBA1 it is able to adenylate Ub about 2 times faster (Figure S2.1) and with UBA6 even 6 times faster (Figure S2.2).

**Figure 5:**
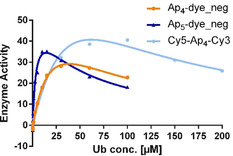
Steady‐state kinetics of UBA1 activated Ub as a function of Ub concentration. The fitting curves represent the nonlinear least squares best fit to the described equation. *K*_i_=50 μm.

Encouraged by these positive results, we determined the velocity of the UBA1 reaction depending on the Ub concentration. Additionally, we compared the herein reported analogues with the best earlier reported doubly labelled ATP analogue (Cy5‐Ap_4_‐Cy3) (for structure see Supporting Information). Interestingly, at high Ub concentration we observed for all three compounds a reaction inhibition (see Supporting Information Figure S2.3‐S2.5), while at low substrate concentration, Michaelis–Menten kinetics were observed. A possible explanation for this observation is that the UBA1‐SH binding site is non‐covalently occupied by a second Ub upon increasing Ub concentration, thus, inhibiting the transfer of adenylated Ub to the active‐site cysteine of UBA1 by forming a thioester with the C‐terminal glycine carboxylate of Ub. Therefore, we fitted our data (Figure 5) to a kinetic model describing such a mode of inhibition^46^ [Eq. 1]:

$$V=\frac{V_{max}}{1+\frac{K_{M}}{\left[S\right]}+\frac{\left[S\right]}{K_{i}}}$$

The high affinity binding site is described by *K*_M_, whereas the inhibitory site, which is in general markedly lower in affinity, is described by *K*_i_. We found for all analogues a *K*_i_ of approximately 50 μm and a *K*_M_ of (4.1±0.5) μm for Ap_5_‐Dye (**11**) which is more than 7 times lower than the *K*_M_ for Ap_4_‐Dye (**6**) (29.1±3.0) μm and almost 20 times lower than the doubly labelled Cy5‐Ap_4_‐Cy3 (76.1±6.7) μm. However, the *K*_M_ for Ub with natural ATP is even lower (0.2 μm) suggesting that our analogues may somewhat interfere with Ub binding.^47^

**Figure 6:**
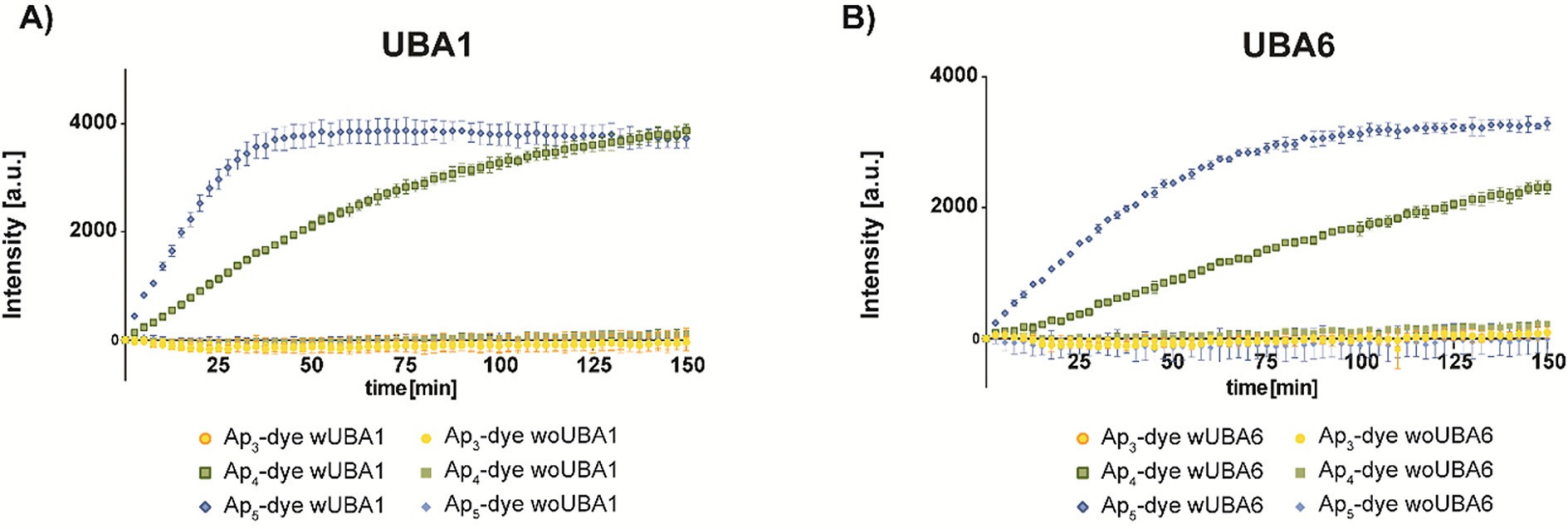
Real‐time autoubiquitination assay with Ap_3_‐Dye, Ap_4_‐Dye and Ap_5_‐Dye=5 μm, MgCl_2_=10 mm, DTT=1.25 mm, UbcH5b=500 nm, E6AP=250 nm, Ub=30 μm and A) UBA1=300 nm, B) UBA6=300 nm. All data represent standard deviation (SD) of triplicates. Excitation *λ*_ex_=360 nm, emission *λ*_em_=450 nm.

Finally, we measured the activation of Ub by UBA1 and UBA6 under conditions, where both thioesters (i.e. UBA1 ∼ Ub and UBA6 ∼ Ub) are unloaded. To do so, we added UbcH5b and E6AP in the same concentration as for the SDS‐PAGE experiment (Figure 4). As shown in Figure 6, again neither UBA1 nor UBA6 are able to activate Ub with Ap_3_‐dye (**5**) as cofactor, while elongation of the phosphate chain rescues activity as already seen in the SDS‐PAGE analysis. Ap_4_‐Dye (**6**) and Ap_5_‐Dye (**11**) are both linearly processed which shows once more the suitability of our setup. Moreover, the extension of the tetraphosphate chain to Ap_5_‐Dye (**11**) increases acceptance of both UBA1 and UBA6 significantly. When all cognate enzymes are present, UBA1 activates Ub approximately 2.9 times faster with Ap_5_‐Dye (**11**) as ATP source compared to Ap_4_‐Dye (**6**). This tendency is also seen with UBA6 where the activation with Ap_5_‐Dye (**11**) is even 4.9 times faster than with Ap_4_‐Dye (**6**).

## Conclusions

In conclusion, we developed and explored novel fluorescently labelled adenosine tri‐, tetra‐, and pentaphosphates. The ATP analogues bear a single acridone‐based dye at the terminus of the phosphate chain. Most importantly, the dye's fluorescence is quenched by the adenine residue of the ATP analogue. Fluorescence is restored upon cleavage of the phosphate chain and dissociation of the dye from the adenosine moiety. Thereby the activity of ATP cleaving enzymes can be followed.

In comparison to our earlier approaches, in this approach only one dye modification is appended to the ATP analogue. This has several advantages. Obviously, the synthesis towards the probes is simplified making these sensors more readily available. Another advantage is the absence of any modification at the nucleobase thereby rendering the analogues to be superiorly processed by the enzymes investigated here. This might be due to the fact that upon usage by the adenylate‐forming enzymes investigated here, the formed reactive adenylated ubiquitin species is identical to that with natural ATP.

By elongation of the phosphate chain, we were able to increase acceptance and reaction velocity significantly and demonstrated this for UBA1 and UBA6 that accept Ap_5_‐Dye (**11**) best. We could also show that the herein presented analogues can be readily used in real‐time assays to follow Ub activation by UBA1 and UBA6. Notably, using the developed disulfonated acridone, the read‐out can be both fluorescence intensity and lifetime. These characteristics make the herein developed ATP analogues versatilely applicable for future uses, for example, in the high‐throughput screening for effectors of E1 enzymes.

## Conflict of interest

The authors declare no conflict of interest.

## Supporting information

As a service to our authors and readers, this journal provides supporting information supplied by the authors. Such materials are peer reviewed and may be re‐organized for online delivery, but are not copy‐edited or typeset. Technical support issues arising from supporting information (other than missing files) should be addressed to the authors.

Supplementary
