# Supplementary material for: Fluorescently Labelled ATP Analogues for Direct Monitoring of Ubiquitin Activation
Source: Chemistry. 2020 Apr 28;26(28):6279–84. doi: 10.1002/chem.202001091 (PMC7317923; doi:10.1002/chem.202001091)
Supplement: Supplementary file 1 — Supplementary [file CHEM-26-6279-s001.pdf]

Chemistry–A European Journal

Supporting Information

Fluorescently Labelled ATP Analogues for Direct Monitoring of Ubiquitin Activation

Daniel Hammler,[a] Katrin Stuber,[a, c] Fabian Offensperger,[c] Martin Scheffner,*[c] Andreas Zumbusch,*[b] and Andreas Marx[a]

1 Contents

1 Experimental Section 2

1.1 General information 2

1.2 General experimental procedures 5

1.2.1 General Procedure 1: Azide reduction 5

1.2.2 General Procedure 2: NHS ester coupling with hydrophilic dyes 5

2 UBA1 & UBA6 real-time assay 6

3 Compound Synthesis and Characterization 10

3.1 Synthesis of γ-modified Adenosine Triphosphate 10

3.1.1 γ-O-(6-Azidohexyl) adenosine-O5'-triphosphate 10

3.1.2 γ-O-(6-Aminohexyl)adenosine-O5'-triphosphate 10

3.2 Synthesis of δ-modified Adenosine Tetraphosphate 11

3.2.1 δ-(6-Azidohexyl)-adenosine-O5'-tetraphosphate 11

3.2.2 δ-(6-Aminohexyl)-adenosine-O5'-tetraphosphate 12

3.3 Synthesis of ε-modified Adenosine Pentaphosphate 12

3.3.1 Diisopropylamino dichlorophosphine (7) 12

3.3.2 Cyclic pyrophosphoryl diisopropylphosphoramidite (8) 13

3.3.3 1-(6-Azido)hexyl phosphoryl cyclotriphosphate (9) 13

3.3.4 ε-(6-Azidohexyl)-adenosine-O5'-pentaphosphate (10) 14

3.3.5 δ-(6-Aminohexyl)-adenosine-O5'-pentaphosphate 14

3.4 Synthesis of Acridone/Quinacridone derivatives 15

3.4.1 Ethyl 6-(9-oxo-9H-acridin-10-yl)-hexanoate (1) 15

3.4.2 6-(9-oxo-2,7-disulfoacridin-10(9H)-yl)hexanoic acid (2.2) 15

3.4.3 S_2Acridone-NHS (13) 16

3.4.4 Ethyl 6-(12-methyl-7,14-dioxo-12,14-dihydroquinolino[2,3-b]acridin-5(7H)-yl)hexanoate (Me-quinacridone-COOEt) (3) 16

3.4.5 6-(12-methyl-7,14-dioxo-2,9-disulfo-12,14-dihydroquinolino[2,3-b]acridin-5(7H)-yl) hexanoic acid (Me–S_2quinacridone–COOH) (4) 17

3.5 Synthesis of fluorescently labelled nucleotides 17

3.5.1 γ-O-(6-(S_2Acridone) amidohexyl) adenosine-O5'-triphosphate (5) 17

3.5.2 δ-O-(6-(S_2Acridone) amidohexyl) adenosine-O5'-tetraphosphate (6) 18

3.5.3 ε-O-(6-(S_2Acridone) amidohexyl) adenosine-O5'-pentaphosphate (11) 18

4 NMR Spectra of all compounds 19

5 Absorption/emission spectra 28

1 Experimental Section

1.1 General information

General Information

All reagents were used without further purification. Dry solvents were obtained from Sigma-Aldrich and used without further purification. Reactions were conducted with exclusion of air and moisture as needed. Anion-exchange chromatography was performed either on an ÄktaPurifier (GE Healthcare, USA) with a DEAE Sephadex™ A-25 (GEHealthcare Bio-SciencesAB, USA) column using a linear gradient (0.1 M – 1.0 M) of triethylammonium bicarbonate buffer (TEAB, pH 7.5) or using a Dionex DNAPac PA-100 22 x 250 mm column using 25 mM Tris-HCL pH = 8 and 5% MeCN as eluent A and 25 mM Tris-HCl pH = 8, 0.5 M $NaClO_4$ and 5% MeCN as eluent B. A linear gradient of 5% B to 40% B was applied with a flow rate of 8 mL/min. Reversed phase high pressure liquid chromatography (RP-HPLC) for the purification of compounds was performed using a Shimadzu unit having LC8a pumps and a Dynamax UV-1 detector. A VP 250/21 NUCLEODUR C18 HTec, 5 µm (Macherey-Nagel, Germany) column and a gradient of MeCN in 50 mM TEAA buffer (pH = 7.0) were used. All compounds purified by RP-HPLC were obtained as their triethylammonium salts after repeated freeze-drying. The ^{1}H NMR signals of triethylammonium are not reported. Analytical RP-HPLC was performed using a Shimadzu Prominence system. A VP 250/4 NUCLEODUR C18 Pyramid, 5 µm (Macherey-Nagel, Germany) column and a gradient of acetonitrile in 50 mM TEAA buffer (pH 7.0) were used.

NMR Spectroscopy and Mass Spectrometry

^{1}H, ^{13}C, ^{19}F and ^{31}P NMR spectra were recorded in commercially available deuterated solvents on a Avance III 400 MHz spectrometer and a AVIII 600 MHz spectrometer (Bruker, Germany). All chemical shifts are given relative to the residual solvent peak and are given in ppm, coupling constants are in Hz.

HR-ESI MS spectra were recorded on a Daltronics microTOF II (Bruker, Germany). The ^{1}H NMR signals of triethylammonium are not listed.

Triethylammonium bicarbonate buffer (TEAB)

1 M TEAB buffer was manufactured by suspending triethylamine (5 mol, 700mL) in water and passing carbon dioxide (from evaporated dry ice) through the mixture until the pH = 7.5. The buffer was diluted to 5 L to give 1 M TEAB. The buffer was diluted to 0.1 M as needed.

Triethylammonium acetate buffer (TEAA)

1 M TEAA buffer was obtained by mixing triethylamine (139 mL, 1 mol) with water and slowly adding acetic acid (57 mL, 1 mol). After cooling to room temperature the pH was adjusted to 7.0 and the buffer was diluted to 1 L to give 1 M TEAA and finally diluted to 50 mM as needed.

1 M $NaHCO_3$ buffer

$NaHCO_3$ (84 g, 1 mol) was dissolved in water and the pH was adjusted to pH = 8.9 or 8.7 as needed using 1 M HCl/NaOH solution. The solution was diluted to 1 L to give 1 M $NaHCO_3$ buffer.

SVPD treatment

The respective mono labelled ATP analogue (100 µM) was incubated with or without SVPD (3.3 mU/µL) in a buffer containing 100 mM NaCl, 100 mM Tris-HCl (pH = 8.7), 15 mM $MgCl_2$ and in a total volume of 30 µL for 45 min at 30 °C.

Absorbance/Fluorescence Spectroscopy

Absorption, emission spectra and fluorescence lifetimes were measured and determined by using standard procedures as depicted in the following. The corresponding mono labelled ATP analogue (0.85 – 1.13 µM) was diluted in 1 x PBS buffer (pH = 7.0) to avoid the inner filter effect (absorbance = 0.1 or lower). All fluorescence experiments at ambient temperatures were performed in 10 mm polystyrene cuvettes. Absorbance spectra were recorded with a Cary 50 Bio UV-Vis spectrophotometer (Varian, USA). Fluorescence emission spectra and fluorescence lifetimes using time correlated single photon counting (TCSPS) were recorded and analysed on a FluoTime300 (PicoQuant, Germany). All fluorophores were excited at 405 nm using a solid-state laser-excitation source (LDH-P-C-405, PicoQuant, Germany). The instrument response function was recorded at the excitation wavelength using a Ludox solution. TCSPC data were analysed using Easy Tau (PicoQuant, Germany).

Photoelectron Spectroscopy in air (PESA)

HOMO energy levels were measured on an instrument for photoelectron spectroscopy at atmospheric pressure that is an open counter equipped with an UV source (3.40 – 6.20 eV; Model AC-2, Riken Keiki, Japan). The organic compounds were separately dissolved in MeOH until saturation and centrifuged. 50 uL of this solution was dropped on a fused silica slide and dried at room temperature overnight. For the measurement, the light intensities were adjusted to an electron background count below 2 cps, and for maximum photon energy below 1000 cps.

E6AP autoubiquitination assay

500 µM of either ATP, **5**, **6** or **11** (final volume 6 uL), 50 mM NaCl and 25 mM Tris-HCl (pH = 7.5) and 0.05 U of rSAP were preincubated at 30°C for 30 min. The mixture was then heated to 65°C for 5 min and cooled to 4°C.

A mixture of 30 nM ubiquitin, 300 nM of UBA1 or UBA6, 500 nM of UbcH5, 250 nM of E6AP in a buffer containing 10 mM $MgCl_2$, 1.25 mM DTT, 50 mM NaCl and 25 mM Tris-HCl (pH = 7.5) was then added to the cooled reaction solution and, in some control reactions as indicated in the respective figures, additional ATP was added which function as (final volume 20 uL, final ATP* conc. = 150 mM). After incubation for 90 min at 30°C the reactions were stopped by adding SDS loading buffer (80 mM Tris-HCl (pH 6.8), 120 mM DTT, 80 mM SDS, 12% glycerol, bromophenol blue) and the total reaction mixtures were analyzed by SDS-PAGE (12.5% gel) followed by staining with Coomassie brilliant blue.

Real-time UBa1 & UBA1 assay

A mixture of the indicated amount of ubiquitin, 300 nM of UBA1 or UBA6, 500 nM of UbcH5, 250 nM of E6AP in a buffer containing 10 mM $MgCl_2$, 1.25 mM DTT, 50 mM NaCl and 25 mM Tris-HCl (pH = 7.5) was preincubated at 30°C for 5 min. The reaction was started with a final concentration of 5 µM of either **5**, **6**, **11** or Cy5-Ap4-Cy3 (final volume 30 uL). Fluorescence intensity was measured every 2.5 min using an infinite F500 plate reader (TECAN, λ_{ex}= 360 ± 35 nm, λ_{em}= 465 ± 35 nm, for Cy5-Ap4-Cy3: λ_{ex}= 535 ± 25 nm, λ_{em}= 590 ± 25 nm). All experiments were performed in triplicates. To avoid photo bleaching effects, the corresponding analogues were dissolved in buffer and set as baseline. The obtained intensity values were subtracted from the first value of each measurement.

Real-time autoubiquitination assay

A mixture of 30 nM ubiquitin, 300 nM of UBA1 or UBA6, 500 nM of UbcH5, 250 nM of E6AP in a buffer containing 10 mM $MgCl_2$, 1.25 mM DTT, 50 mM NaCl and 25 mM Tris-HCl (pH = 7.5) was preincubated at 30°C for 5 min. The reaction was started with a final concentration of 5 µM of either **5**, **6** or **11** (final volume 30 uL). Fluorescence intensity was measured every 2.5 min using an infinite F500 plate reader (TECAN, λ_{ex}= 360 ± 35 nm, λ_{em}= 465 ± 35 nm). All experiments were performed in triplicates. To avoid photo bleaching effects, the corresponding analogues were dissolved in buffer and set as baseline. The obtained intensity values were subtracted from the first value of each measurement.

Expression and purification of proteins

His-tagged human E1 enzyme (UBA1) was expressed in *E.coli* BL21 RIL DE3 and purified via HisTrap FF (5 mL) followed by size exclusion chromatography[1]. Ubiquitin was expressed in E. coli BL21 DE3 and purified by HisTrap FF (5 mL) followed by cation exchange chromatography[2].

His-tagged UbcH5b was expressed in *E. coli* BL21 DE3 and purified via Ni-NTA affinity chromatography[3]. Fractions containing UbcH5b were pooled, dialyzed against 25 mM Tris-HCl pH = 7.5, 50 mM NaCl, 0.1 mM DTT and stored at −80 °C.

His-tagged E6AP was expressed in *E. coli* Rosetta DE3 at 20°C overnight. Cell pellets derived from 1 L bacterial culture were resuspended in 30 mL of 25 mM Tris-HCl pH 7.5, 50 mM NaCl, 0.1% Triton, 1 mM DTT, 1 µg/mL aprotinin and leupeptin, and 100 µM Pefabloc. After sonication and centrifugation (15 000×g, 4°C, 15 min), the supernatant was loaded onto a HisTrap FF (5 mL), washed with 8 column volumes of buffer A (25 mM Tris-HCl pH 7.5, 50 mM NaCl, 1 mM DTT) followed by a gradient of 20 column volumes to 100% buffer B (25 mM Tris-HCl, 50 mM NaCl, 500 mM imidazole, pH = 7.5). Fractions containing E6AP were pooled and subjected to a second purification step by anion exchange chromatography (HiTrapTM Q HP, 1 mL column), using a gradient from 0 to 50% buffer B in 20 column volumes (buffer A: 25 mM Tris-HCl pH 7.5, 50 mM NaCl, 1 mM DTT; buffer B: 25 mM Tris-HCl pH 7.5, 1 M NaCl, 1 mM DTT). 4 mL Amicon filter devices with a cutoff of 10 kDa were used for buffer exchange to 25 mM Tris-HCl pH = 7.5, 50 mM NaCl, 1 mM DTT and to concentrate the sample[4].

UBA6

UBA6 (human), (recombinant) was bought from Enzo Life Sciences GmbH and used without further purification.

1.2 General experimental procedures

1.2.1 General Procedure 1: Azide reduction

The azide modified nucleotide (1 equiv, approx. 3 mM) was dissolved in water/methanol/triethylamine (2:2:1) and tris-(2-carboxyethyl)-phosphine hydrochloride (5 equiv) was added and stirred at 4°C for 12-20 h. The solvents were evaporated under reduced pressure. The compound was purified by RP-HPLC. Fractions containing the product were evaporated and the product repeatedly freeze-dried from water to give the free amine.

1.2.2 General Procedure 2: NHS ester coupling with hydrophilic dyes

The nucleotide containing a free amine (1 equiv, 1 – 5 mM) was dissolved in $NaHCO_3$ (adjusted to pH = 8.7, final concentration 0.1 M) and the appropriate NHS ester (1.5 – 4 equiv) was added. Immediately after addition the pH was readjusted to pH = 8.7 and monitored for the next 30 minutes. The solution was stirred at room temperature for 1 hour until complete conversion was achieved. The compound was purified by IEX (Dionex DNAPac PA-100 column) followed by RP-HPLC. The solvent was evaporated and the product repeatedly freeze-dried from water to give the labelled nucleotide with triethylammonium as the counter ion.

2 UBA1 & UBA6 real-time assay

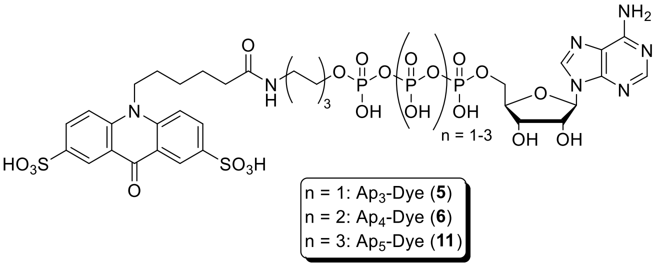

n = 1: Ap_3-Dye (**5**)
n = 2: Ap_4-Dye (**6**)
n = 3: Ap_5-Dye (**11**)

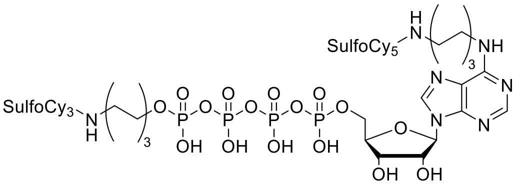

Cy5-Ap_4-Cy3

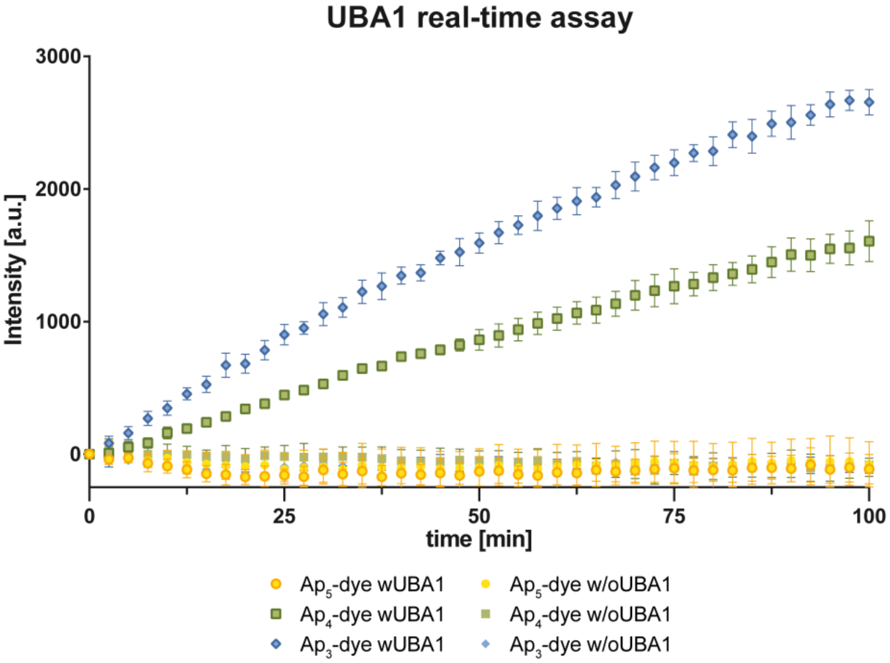

Figure S2.1: Real-time UBA1 assay with Ap_3-Dye (**5**), Ap_4-Dye (**6**) and Ap_5-Dye (**11**) = 5 µM, $MgCl_2$ = 10 mM, DTT = 1.25 mM, Ub = 10 µM and UBA1 = 300 nM. All data represent standard deviation (SD) of triplicates. Excitation λ_{ex} = 360 nm, emission λ_{em} = 450 nm.

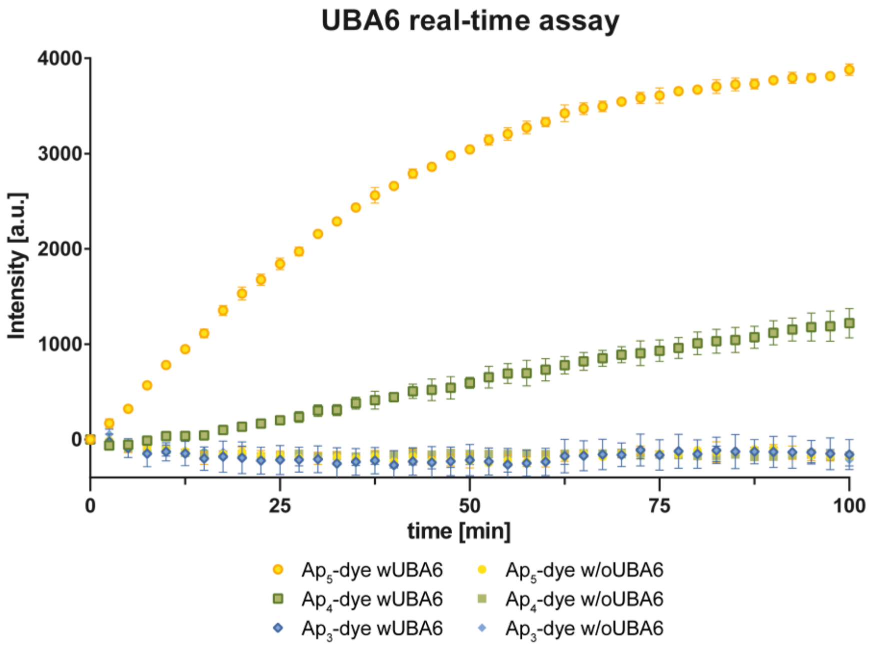

Figure S2.2: Real-time UBA6 assay with Ap3-Dye (**5**), Ap4-Dye (**6**) and Ap5-Dye (**11**) = 5 µM, $MgCl_2$ = 10 mM, DTT = 1.25 mM, Ub = 30 µM and UBA6 = 300 nM. All data represent standard deviation (SD) of triplicates. Excitation λ_{ex} = 360 nm, emission λ_{em} = 450 nm.

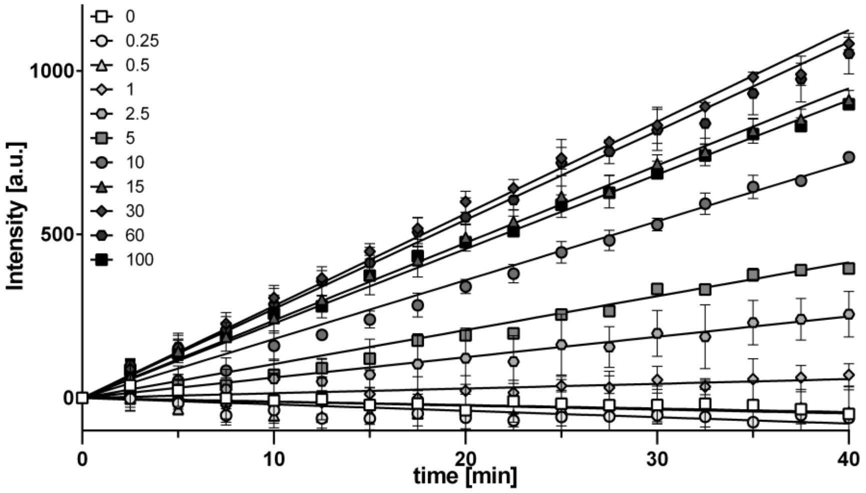

Figure S2.3: Real-time UBA1 assay with Ap4-Dye (**6**) = 5 µM, $MgCl_2$ = 10 mM, DTT = 1.25 mM, Ub = x µM and UBA1 = 300 nM. All data represent standard deviation (SD) of triplicates. Excitation λ_{ex} = 360 nm, emission λ_{em} = 450 nm.

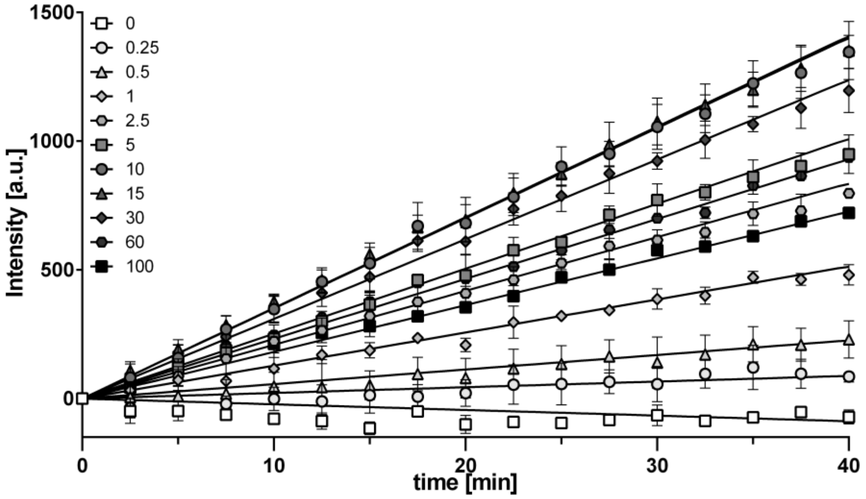

Figure S2.4: Real-time UBA1 assay with Ap$_5$-Dye (**11**) = 5 µM, $MgCl_2$ = 10 mM, DTT = 1.25 mM, Ub = x µM and UBA1 = 300 nM. All data represent standard deviation (SD) of triplicates. Excitation λ_{ex} = 360 nm, emission λ_{em} = 450 nm.

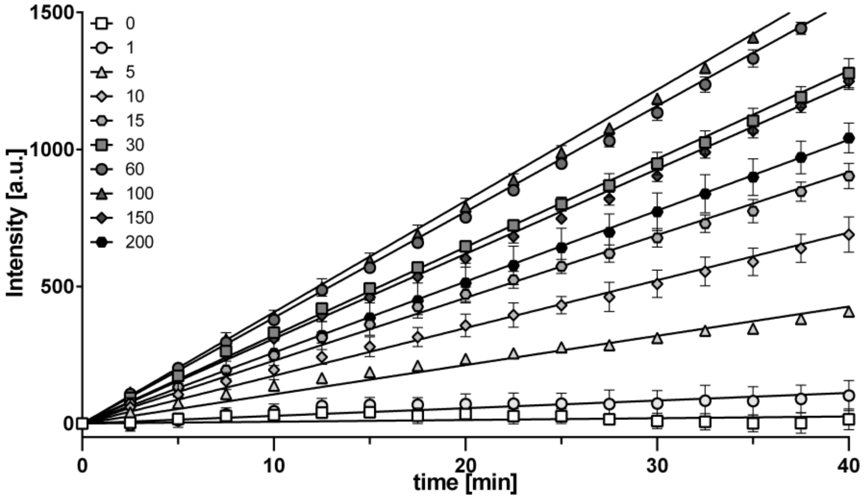

Figure S2.5: Real-time UBA1 assay with Cy5-Ap$_4$-Cy3 (structure shown at page 6) = 5 µM, $MgCl_2$ = 10 mM, DTT = 1.25 mM, Ub = x µM and UBA1 = 300 nM. All data represent standard deviation (SD) of triplicates. Excitation λ_{ex} = 360 nm, emission λ_{em} = 450 nm.

	Ap$_4$-Dye		Ap$_5$-Dye		Cy5-Ap$_4$-Cy3	
Ub conc.	slope	SD	slope	SD	slope	SD
0	-1.08	0.276	-2.198	0.1593	0.6374	0.1352
0.25	-1.959	0.08736	2.211	0.28		
0.5	-1.18	0.2048	5.654	0.2593		
1	1.444	0.1387	12.84	0.1656	2.79	0.1961
2.5	6.237	0.246	20.92	0.2687		
5	10.38	0.1143	25.22	0.3241	10.67	0.112
10	18	0.3061	35.05	0.3274	17.43	0.2164
15	23.7	0.2058	35.21	0.4103	22.93	0.2164
30	28.16	0.311	30.96	0.2455	32.19	0.1779
60	27.22	0.2455	23.26	0.1565	38.63	0.1857
100	22.77	0.1816	18.16	0.1179	40.61	0.1761
150					30.95	0.133
200					25.89	0.2139

Table S2.1*: Linear fit of the initial reaction speed with different Ub concentration.*

3 Compound Synthesis and Characterization

3.1 Synthesis of γ-modified Adenosine Triphosphate

3.1.1 γ-O-(6-Azidohexyl) adenosine-O5'-triphosphate (15)

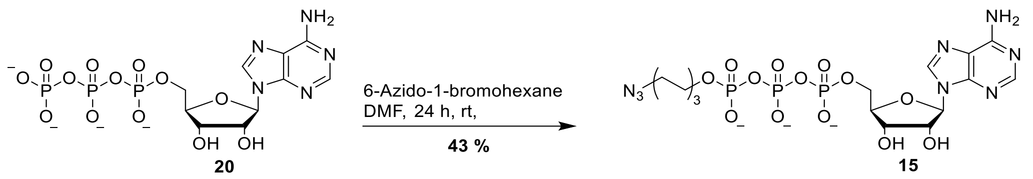

γ-O-(6-Azidohexyl) adenosine-O5'-triphosphate (**15**) was prepared as previously described by Lee *et al.*[5].

Adenosinetriphosphate (**20**) (150 µmol, 1.0 equiv) was converted into its tetrabutylammonium salt by passing it through a column containing Chelex100 preequilibrated with Bu_4NBr. The tetrabutylammonium triphosphate was freeze-dried overnight and then dissolved in DMF. 6-Azido-1-bromohexane[6] was prior dissolved in dry DMF (c = 0.5 M) and stored for several days over molecular sieve. 6-Azido-1-bromohexane (0.9 mL, 450 µmol, 3 equiv) was added to the dried triphosphate and stirred at room temperature for 24 hours, the solvent was evaporated under reduced pressure and the crude product was purified by RP-HPLC which yields γ-O-(6-azidohexyl) adenosine-O5'-triphosphate (**15**) with little impurities which could be removed by anion-exchange chromatography (65 µmol, 43%).

^{1}H NMR (400 MHz, MeOD-d_4): δ = 8.52 (s, 1H, H-8), 8.14 (s, 1 H H-2), 6.03 (d, J = 5.5 Hz, 1H, H-1'), 4.67 (t, J = 5.3 Hz, 1H, H-2'), 4.51 (t, J = 4.2 Hz, 1H, H-3'), 4.30 – 4.22 (m, 1H, H-4'), 4.22 – 4.15 (m, 2H, H-5'), 3.94 (q, J = 6.5 Hz, 2H P-γ-O–C**H**$_2$), 3.19 (t, J = 6.8 Hz, 2H, N_3–C**H**$_2$), 1.56 (p, J = 6.7 Hz, 2H, C**H**$_2$-linker), 1.48 (q, J = 6.8 Hz, 2H, C**H**$_2$-linker), 1.31 (dt, J = 6.8, 3.5 Hz, 4H, 2x C**H**$_2$-linker).

^{31}P NMR (162 MHz, MeOD-d_4): δ = -11.29 (d, J = 18.5 Hz), -11.76 (d, J = 18.3 Hz), -23.25 (t, J = 18.7 Hz).

HR MS (ESI, neg. mode): m/z: calculated for $C_{18}H_{28}F_3N_7O_{14}P_3^-$ [M-H]$^-$: 631.0827,
found: 631.0845 [M-H]$^-$, deviation: 2.8 ppm.

3.1.2 γ-O-(6-Aminohexyl)adenosine-O5'-triphosphate (16)

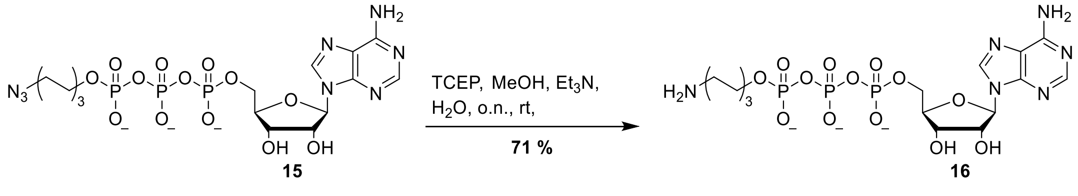

γ-O-(6-Aminohexyl) adenosine-O5'-triphosphate (**16**) was synthesized from γ-O-(6-azidohexyl) adenosine-O5'-triphosphate (**15**) (65 *µ*mol, 1.0 equiv) according to the general procedure 1 and was obtained as a white powder (46 µmol, 71%).

^{1}H NMR (400 MHz, D_2O): δ = 8.58 (s, 1H, H-8), 8.27 (s, 1 H H-2), 6.16 (d, *J* = 5.9 Hz, 1H, H-1'), 4.83 (t, *J* = 5.6 Hz, 1H, H-2'), 4.61 (dd, *J* = 5.1, 3.5 Hz, 1H, H-3'), 4.44 (p, *J* = 2.9 Hz, 1H, H-4'), 4.29 (td, *J* = 5.5, 4.9, 3.1 Hz, 2H, H-5'), 3.95 (q, *J* = 6.5 Hz, 2 H, P-γ-O–C**H**$_2$), 3.00 (t, *J* = 7.4 Hz, 2 H, NH_2-C**H**$_2$), 1.63 (q, *J* = 6.8 Hz, 2H, NH_2-CH_2-C**H**$_2$), 1.57 (q, *J* = 6.8 Hz, 2H, C**H**$_2$-linker), 1.33 (m, 4H, 2x C**H**$_2$-linker).

^{13}C NMR (101 MHz, D_2O): δ = 155.50, 152.79, 149.18, 140.06, 118.58, 86.85, 84.15 (d, *J* = 9.1 Hz), 74.38, 70.52, 66.55 (d, *J* = 6.1 Hz), 65.36 (d, *J* = 5.8 Hz), 29.31 (d, *J* = 7.2 Hz), 26.55, 25.00, 24.28.

^{31}P NMR (162 MHz, D_2O): δ = -11.29 (d, *J* = 18.5 Hz), -11.76 (d, *J* = 18.3 Hz), -23.25 (t, *J* = 18.7 Hz).

HR MS (ESI, neg. mode): m/z: calculated for $C_{18}H_{28}F_3N_7O_{14}P_3^-$ $[M-H]^-$: 605.0933,
found: 605.0944 $[M-H]^-$, deviation: 1.8 ppm.

3.2 Synthesis of δ-modified Adenosine Tetraphosphate

3.2.1 δ-(6-Azidohexyl)-adenosine-O5′-tetraphosphate (17)

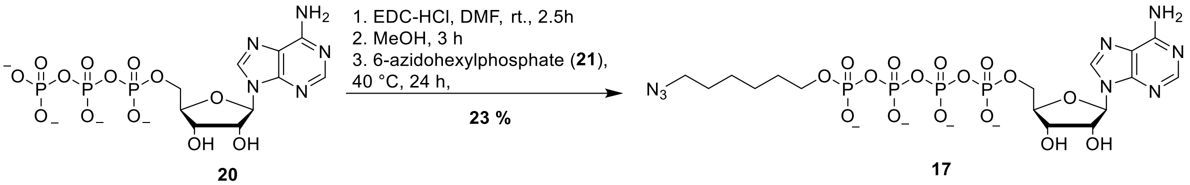

δ-(6-Azidohexyl)-adenosine-O5′-tetraphosphate (**17**) was synthesized as previously described by Hacker *et al.*[7].

Adenosine triphosphate (**20**) (200 µmol, 1 equiv) was converted into its tetrabutylammonium salt by passing it through a preequilibrated CHELEX 100 cation-exchange resin with tetrabutylammonium bromide. Compound **20** and 6-azidohexylphosphate[8] (**21**) (326 mg, 1.40 mmol, 7.0 equiv) were separately lyophilized overnight, subsequently dried in high vacuum for several hours and dissolved in dry DMF (2 mL and 3 mL). 1-Ethyl-3-(3-dimethylaminopropyl) carbodiimid hydrochloride (EDC•HCl) (115 mg, 0.6 mmol, 3 equiv) were added to compound **20** and the solution was stirred at room temperature for 2.5 hours. Methanol (65 µL, 1.6 mmol, 8 equiv) was added and after 3 hours Et_3N (550 µL, 401 mg, 4 mmol, 20 equiv) and 6-azidohexylphosphate (**21**) were added and stirred at 40 °C for 24 hours. The solvent was evaporated and the reaction was quenched with 0.1 M TEAB buffer (15 mL). The product was purified by anion-exchange chromatography (DEAE Sephadex™ A-25 column) and RP-HPLC. Fractions containing the product were evaporated and the product repeatedly freeze-dried from water to give δ-(6-azidohexyl)-N^6-(6-trifluoroacet-amido-hexyl) adenosine-O5′-tetraphosphate (**17**) as a white solid (45.6 µmol, 23%).

^{1}H NMR (400 MHz, MeOD): δ = 8.72 (s, 1H, H-8′), 8.31 (s, 1H, H-2′), 6.11 (d, J = 5.8 Hz, 1H, H-1), 4.71 (t, J = 5.5 Hz, 1H, H-2), 4.62 (dd, J = 5.0, 2.9 Hz, 1H, H-3), 4.40–4.32 (m, 1H, H-5), 4.30–4.23 (m, 2H, H-4 and H-5), 4.03 (q, J = 6.5 Hz, 2H, P-δ-O-C**H**$_2$), 3.27 (t, J = 6.9 Hz, 2H, N_3-C**H**$_2$), 1.64 (p, *J* = 6.6 Hz, 2H, C**H**$_2$-linker), 1.56 (p, *J* = 6.9 Hz, 2H C**H**$_2$-linker), 1.46–1.37 (m, 4H, 2 × C**H**$_2$ linker).

^{31}P NMR (162 MHz, D_2O): δ = -11.08 (m, 1P), -11.58 (m, 1P), -23.20 (m, 2P).

HR MS (ESI, neg. mode): m/z: calculated for $C_{16}H_{27}N_8O_{16}P_4^-$ $[M-H]^-$: 711.0490,
found: 711.0512 $[M-H]^-$, deviation: 3.1 ppm.

3.2.2 δ-(6-Aminohexyl)-adenosine-O5'-tetraphosphate (18)

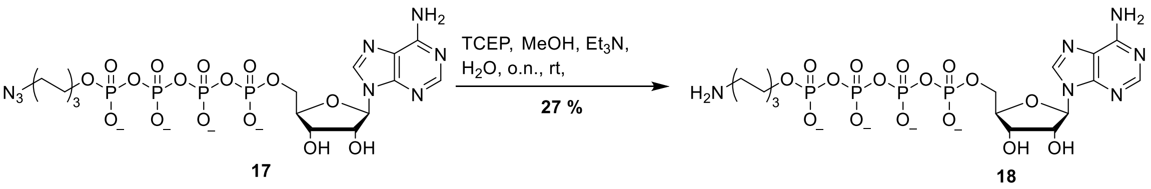

γ-O-(6-Aminohexyl) adenosine-O5'-tetraphosphate (**18**) was synthesized from γ-O-(6-azidohexyl) adenosine-O5'-tetraphosphate (**17**) (30 *μ*mol, 1.0 equiv) according to the general procedure 1 and was obtained as a white powder (8 µmol, 27%).

^{1}H NMR (400 MHz, D_2O): δ = 8.58 (s, 1H, H-8), 8.28 (s, 1 H H-2), 6.16 (d, *J* = 6.2 Hz, 1H, H-1'), 4.84 (t, *J* = 5.8 Hz, 1H, H-2'), 4.62 (t, *J* = 4.1 Hz, 1H, H-3'), 4.42 (p, *J* = 2.9 Hz, 1H, H-4'), 4.36 – 4.16 (m, 2H, H-5'), 3.99 (q, *J* = 6.6 Hz, 2 H, P-δ-O-C**H**$_2$), 3.00 (t, *J* = 7.3 Hz, 2 H, NH_2-C**H**$_2$), 1.64 (m, 4H, 2x C**H**$_2$-linker), 1.39 (m, 4H, 2x C**H**$_2$-linker).

^{31}P NMR (162 MHz, D_2O): *δ* = -10.43 – -11.15 (m, 1P), -11.15 – -11.53 (m, 1P), -22.95 – -23.58 (m, 2P).

HR MS (ESI, neg. mode): m/z: calculated for $C_{16}H_{29}N_6O_{16}P_4^-$ [M-H]$^-$: 685.0596,
found: 685.0611 [M-H]$^-$, deviation: 2.2 ppm.

3.3 Synthesis of ε-modified Adenosine Pentaphosphate

3.3.1 Diisopropylamino dichlorophosphine (7)

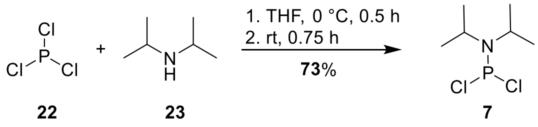

Diisopropylamino dichlorophosphine (**7**) was synthesized as previously described by Song *et al.*[9].

N,*N*-Diisopropylamine (**23**) (7.39 g, 10.30 mL, 73 mmol, 2.1 eq) was added drop-wise to an ice-cooled solution of phosphorus trichloride (**22**) (4.80 g, 3.06 mL, 35 mmol, 1.0 eq) in dry THF (50 mL). After 30 min, the ice bath was removed and the reaction was warmed to room temperature and stirred for another 45 min. The white precipitate was removed by filtration under N_2 and washed with dry THF (1 x 5 mL). The solvent was evaporated and the remaining solution distilled (1.5 mbar, 40 °C) to yield diisopropylamino dichlorophosphine (**7**) (5.17 g, 25.6 mmol, 73%).
For short-term usage, the product was stored at 7 °C otherwise it was stored at -23 °C.

^{1}H NMR (400 MHz, $CDCl_3$): *δ* = 3.93 (dhept, *J* = 12.5, 6.8 Hz, 2H, 2x C**H**(CH_3)$_2$), 1.28 (d, *J* = 6.9 Hz, 12H, 4x C**H**$_3$).

^{31}P NMR (400 MHz, $CDCl_3$): *δ* = 169.51 (s).

3.3.2 Cyclic pyrophosphoryl diisopropylphosphoramidite (8)

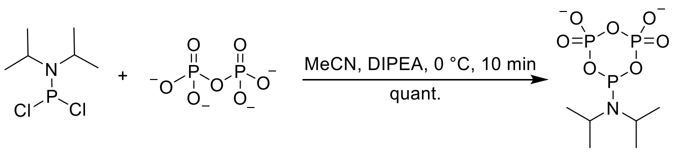

7 **24** **8**

Cyclic pyrophosphoryl diisopropylphosphoramidite (**8**) and the two next steps (compound **9** and **10**) are synthesized according to Singh *et al.*[10].

Pyrophosphate*TBA (**24**) (274 mg, 500 µmol, 1.0 eq) was freeze-dried overnight, coevaporated three times with dry MeCN (1.5 mL) and dissolved in dry MeCN (4.5 mL). DIPEA (255 µL, 193.3 mg, 1.5 mmol, 3.1 eq) was added and the solution cooled to 0 °C. Diisopropylamino dichlorophosphine (**7**) (95.1 µL, 101 mg, 500 µmol, 1.0 eq) was dissolved in dry MeCN (3.5 mL) and added drop-wise to the reaction. The solution was stirred for 10 minutes to yield cyclic pyrophosphoryl diisopropylphosphoramidite (**8**) with an assumed concentration of 62.5 mM.

^{31}P NMR (162 MHz, DMF-d7): δ = 129.02 (t, J = 24.5 Hz, 1P), -19.40 (d, J = 24.5 Hz, 2P).

HR MS (ESI, neg. mode): m/z: calculated for $C_6H_{15}NO_7P_3^-$ [M-H]$^-$: 306.0056,
found: 306.0068 [M-H]$^-$, deviation: 3.9 ppm.

3.3.3 1-(6-Azido)hexyl phosphoryl cyclotriphosphate (9)

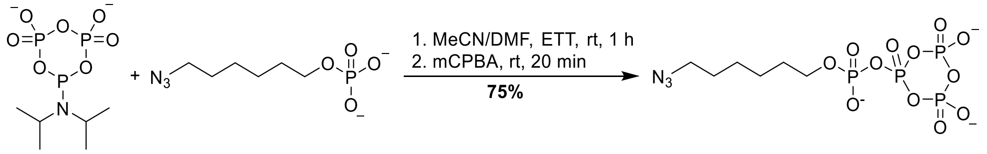

8 **21** **9**

Freshly synthesized cyclic pyrophosphoryl diisopropylphosphoramidite (**8**) (62.5 mM in MeCN, 1.92 mL, 120 µmol, 1.2 eq) and 6-azidohexyl phosphate*TBA (**21**) (0.36 mM in DMF, 277 µL, 100 µmol, 1.0 eq) were stirred with 5-(ethylthio)-1H-tetrazole (ETT) (750 µM in MeCN, 333 µL, 250 µmol, 2.5 eq) for 1 hour. *m*-Chloroperoxybenzoic acid (*m*CPBA) (26 mg, 77% absolute, 150 µmol, 1.5 eq) was added and the solution stirred for another 20 minutes to yield 1-(6-azido)hexyl phosphoryl cyclotriphosphate (**9**) (75 µmol, 75%, estimated by NMR).
The yield was determined using the NMR spectrum which showed 25% contamination. The product was stored in the freezer at −80 °C and used for further synthesis without purification.

^{1}H NMR (400 MHz, DMF-*d7*): δ = 2.99 (dt, J = 2.9, 2.2 Hz, 2H, N_3-C**H**$_2$), 2.87 (dt, J = 3.0, 2.3 Hz, 2H, PO_4-C**H**$_2$), 2.07 (q, J = 5.7, 2.7 Hz, 8H, N_3-CH_2-C**H**$_2$-C**H**$_2$).

^{31}P NMR (162 MHz, DMF-*d7*): δ = -13.45 (d, J = 18.7 Hz, 1P), -25.08 – -25.59 (m, 2P), -34.51 (q, J = 21.4 Hz, 1P).

Unoxidized:
HR MS (ESI, neg. mode): m/z: calculated for $C_6H_{15}NO_{11}P_4^-$ [M-H]$^-$: 427.9587,
found: 427.9573 [M-H]$^-$, deviation: 3.3 ppm.

Oxidized:
HR MS (ESI, neg. mode): m/z: calculated for $C_6H_{15}NO_{12}P_4^-$ [M-H]$^-$: 443.9522,
found: 443.9645 [M-H]$^-$, deviation: 3.9 ppm.

3.3.4 ε-(6-Azidohexyl)-adenosine-O5'-pentaphosphate (10)

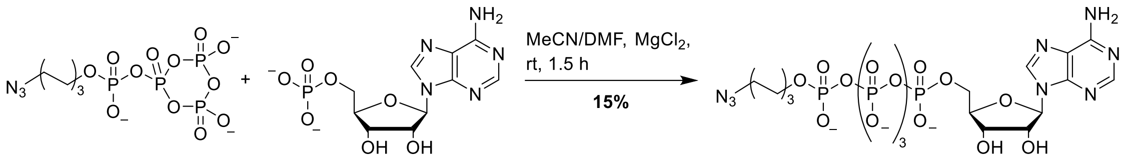

AMP*1.5 TBA (75 µmol, 1.0 eq) was freeze-dried overnight and then coevaporated once with MeCN (1.5mL). 1-(6-Azido)hexyl phosphoryl cyclotriphosphate (**9**) (30 mM in DMF/MeCN, 2.5 mL, 75 µmol, 1.0 eq) and magnesium chloride (14.3 mg, 150 µmol, 2 eq) were added to the reaction. The solution was stirred for 1.5 hours. Diethyl ether was added to the reaction to precipitate the product, the suspension was centrifuged, the pellet dissolved in H_2O and purified in two fractions by IEX (Hi-Trap 5mL) and RP-HPLC according to the general procedures to yield ε-(6-azido-hexyl)-adenosine-O5'-pentaphosphate (**10**) (11.25 µmol, 15%).

^{1}H NMR (400 MHz, D_2O): δ = 8.53 (s, 1H, H-8'), 8.22 (s, 1H, H-2'), 6.07 (d, J = 5.8 Hz, 1H, H-1), 4.78 (t, J = 5.5 Hz, 1H, H-2), 4.56 (dd, J = 5.0, 2.9 Hz, 1H, H-3), 4.34 (q, J = 2.9 Hz, 1H, H-4), 4.30 – 4.09 (m, 2H, H-5), 3.91 (q, J = 6.5 Hz, 2H, P-ε-O-C**H**$_2$), 3.22 (t, J = 6.9 Hz, 2H, N_3-C**H**$_2$), 1.64 – 1.47 (m, 4H, 2 × C**H**$_2$ linker), 1.34 – 1.23 (m, 4H, 2 × C**H**$_2$ linker).

^{31}P NMR (162 MHz, D_2O): *δ* = -10.90 (d, J = 16.3 Hz, 1P), -11.55 (d, J = 16.9 Hz, 1P), -22.63 – -24.09 (m, 3P).

HR MS (ESI, neg. mode): m/z: calculated for $C_{16}H_{28}N_8O_{19}P_5^-$ [M-H]$^-$: 791.0153,
found: 791.0161 [M-H]$^-$, deviation: 1.0 ppm.

3.3.5 δ-(6-Aminohexyl)-adenosine-O5'-pentaphosphate (19)

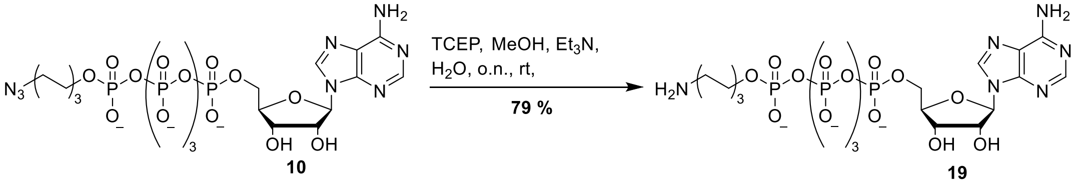

γ-O-(6-Aminohexyl) adenosine-O5'-pentaphosphate (**19**) was synthesized from γ-O-(6-azidohexyl) adenosine-O5'-pentaphosphate (**10**) (10 *µ*mol, 1.0 equiv) according to the general procedure 1 and was obtained as a white powder (7.9 µmol, 79%).

^{1}H NMR (400 MHz, D_2O): δ = 8.59 (s, 1H, H-8), 8.29 (s, 1 H H-2), 6.16 (d, *J* = 6.4 Hz, 1H, H-1'), 4.85 (t, *J* = 5.8 Hz, 1H, H-2'), 4.71 – 4.61 (m, 1H, H-3'), 4.44 (t, *J* = 2.9 Hz, 1H, H-4'), 4.39 – 4.15 (m, 2H, H-5'), 4.02 (q, *J* = 6.6 Hz, 2 H, P-ε-O-**CH**$_2$), 3.02 (t, *J* = 7.3 Hz, 2 H, NH_2-C**H**$_2$), 1.77 – 1.63 (m, 4H, 2x C**H**$_2$-linker), 1.50 – 1.41 (m, 4H, 2x C**H**$_2$-linker).

^{31}P NMR (162 MHz, D_2O): *δ* = -10.80 (d, *J* = 17.1 Hz, 1P), -11.40 (d, *J* = 17.8, 16.8 Hz, 1P), -22.53 – -23.95 (m, 3P).

HR MS (ESI, neg. mode): m/z: calculated for $C_{16}H_{30}N_6O_{19}P_5^-$ [M-H]$^-$: 765.0248,
found: 765.0254 [M-H]$^-$, deviation: 0.8 ppm.

3.4 Synthesis of Acridone/Quinacridone derivatives

3.4.1 Ethyl 6-(9-oxo-9H-acridin-10-yl)-hexanoate (1)

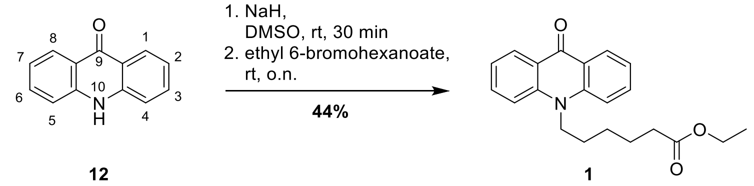

6-(9-oxo-9H-acridin-10-yl)-hexanoate (**1**) was synthesized according to Smith *et al.*[11].

9-Oxo-9,10-dihydro-acridine (**12**) (2.44 g, 12.5 mmol, 1 equiv) was suspended in DMSO (15 mL) and sodium hydride (95%, 332 mg, 13.13 mmol, 1.05 equiv) was added in portions. After effervescence ceased (30 minutes), ethyl 6-bromohexanoate (2.45 mL, 3.07 g, 13.75 mmol, 1.1 equiv) was added and the reaction stirred overnight. The reaction was quenched with water and extracted with EE (3 x 30 mL). The organic phase was washed with brine (30 mL) and dried with $MgSO_4$. The crude product was purified by flash chromatography (15% EE in DCM) and the fractions with the pure product concentrated to yield ethyl 6-(9-oxo-9H-acridin-10-yl)-hexanoate (**1**) as a yellow powder (1.86 g, 5.51 mmol, 44%).

^{1}H NMR (400 MHz, $CDCl_3$): δ = 8.60 (dd, J = 8.0, 1.8 Hz, 2H, Ar1, Ar8), 7.74 (ddd, J = 8.7, 6.9, 1.7 Hz, 2H, Ar3, Ar6), 7.50 (d, J = 8.7 Hz, 2H, Ar4, Ar5), 7.31 (td, J = 7.5, 1.5 Hz, 2H, Ar2, Ar7), 4.37 (t, *J* = 7.8 Hz, 2H, N-C**H**$_2$), 4.16 (q, J = 7.1 Hz, 2H, COO-C**H**$_2$-CH$_3$), 2.39 (t, J = 7.3 Hz, 2H, CO-C**H**$_2$), 1.98 (p, J = 8.6, 7.6 Hz, 2H, N-CH$_2$-C**H**$_2$), 1.80 (p, J = 7.3 Hz, 2H, CO-CH$_2$-C**H**$_2$), 1.69 – 1.52 (m, 2H, linker), 1.27 (t, J = 7.1 Hz, 2H, COO-CH$_2$-C**H**$_3$).

3.4.2 6-(9-oxo-2,7-disulfoacridin-10(9H)-yl)hexanoic acid (2.2)

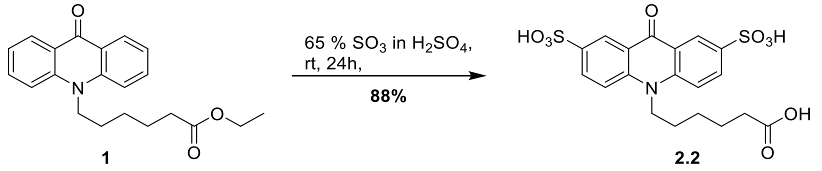

6-(9-oxo-2,7-disulfoacridin-10(9H)-yl)hexanoic acid (**2.2**) was synthesized according to a modified procedure by Smith *et al.*[11].

Ethyl 6-(9-oxo-9H-acridin-10-yl)-hexanoate (**1**) (100 mg, 293 µmol, 1 equiv) was dissolved in fuming sulfuric acid (65%, 1 mL) and stirred at room temperature for 20 hours. The crude product was carefully poured on ice (20 g) and purified with RP-MPLC (solvent A: water + 0.1% TFA, Solvent B: MeOH + 0.1% TFA, linear gradient from 5 - 40% B). Fractions containing the product were evaporated and the product repeatedly freeze-dried from water to give 6-(9-oxo-2,7-disulfoacridin-10(9H)-yl)hexanoic acid (S_2Acridone-COOH) (**2.2**) as a yellow powder (112 mg, 257.8 µmol, 88%).

^{1}H NMR (400 MHz, D_2O): δ = 8.53 (p, J = 2.1 Hz, 2H, Ar1, Ar8), 7.95 (d, J = 9.0 Hz, 2H, Ar3, Ar6), 7.43 (dq, J = 8.9, 2.5 Hz, 2H, Ar4, Ar5), 3.97 (d, J = 8.3 Hz, 2H, N-C**H**$_2$), 2.24 (t, *J* = 7.0 Hz, 2H, CO-C**H**$_2$), 1.49 (dq, J = 14.4, 7.0 Hz, 4H, N-CH$_2$-C**H**$_2$-CH$_2$-C**H**$_2$), 1.31 (d, J = 7.5 Hz, 2H, N-CH$_2$-CH$_2$-C**H**$_2$).

HR MS (ESI, neg. mode): m/z: calculated for $C_{19}H_{18}NO_9S_2^-$ [M-H]$^-$: 468.0417,
found: 468.0433 [M-H]$^-$, deviation: 3.4 ppm.

3.4.3 S2Acridone-NHS (13)

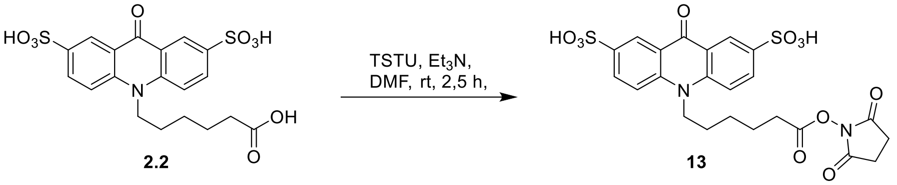

S2Acridone-COOH (**2.2**) (5.0 mg, 10.66 µmol, 1.0 equiv) was dissolved in dry DMF (2 mL), TEA (7.3 µL, 5.4 mg, 53.3 µmol, 5.0 equiv) and TSTU (4.8 mg, 15.99 µmol, 1.5 equiv) were added and stirred for 2.5 hours at room temperature.

The activated dye (S2Acridone-NHS) (**13**) was then directly transferred in several portions to a free amine without further purification.

HR MS (ESI, neg. mode): m/z: calculated for $C_{23}H_{21}N_2O_{11}S_2^-$ [M-H]$^-$: 565.0581,
found: 565.0561 [M-H]$^-$, deviation: 3.5 ppm.

3.4.4 Ethyl 6-(12-methyl-7,14-dioxo-12,14-dihydroquinolino[2,3-b]acridin-5(7H)-yl)hexanoate (Me-quinacridone-COOEt) (3)

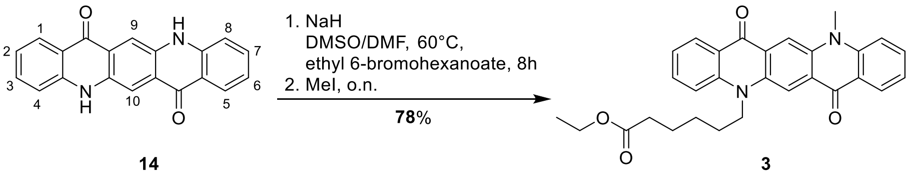

Me−S2quinacridone−COOEt (**4**) was synthesized according to a modified procedure by Smith et al.[11].

Quinacridone (**14**) (1.56 g, 5.00 mmol, 1.0 equiv) was dissolved in dry DMF (15mL) and dry DMSO (15mL), followed by sodium hydride (NaH) (6 mmol, 152 mg, 1.2 equiv) and the reaction was heated to 60 °C. After effervescence ceased (40 min), ethyl 6-bromohexanoate (890 µL, 1.115 g, 5 mmol, 1.0 equiv) was added and the reaction stirred for 8 h. Methyliodid (778 µL, 1.77 g, 12.5 mmol, 2.5 equiv) was subsequently added and the reaction was stirred overnight. The reaction was quenched with water 300mL and the solid filtered. The crude product was purified by flash chromatography (15% EE in DCM) to yield Me−quinacridone−COOEt (**3**) as an orange powder (1.825 g, 3.9 mmol, 78%).

^{1}H NMR (400 MHz, $CDCl_3$): δ = 8.61 (s, 1H, Ar9), 8.58 (s, 1H, Ar10), 8.54 – 8.40 (m, 2H, Ar1, Ar5), 7.64 (m, 2H, Ar3, Ar7), 7.41 (m, 2H, Ar4, Ar8), 7.24 – 7.10 (m, 2H, Ar2, Ar6), 4.44 (t, J = 8.3 Hz, 2H, N-CH_2), 4.16 (qd, J = 7.2, 1.9 Hz, 2H, COO-C**H**$_2$-CH_3), 3.97 (d, J = 1.8 Hz, 3H, Me), 2.40 (ddd, J = 7.4, 5.6, 1.8 Hz, 2H, CO-C**H**$_2$), 2.00 (q, J = 10.8, 7.9 Hz, 2H, N-CH_2-C**H**$_2$), 1.93 – 1.71 (m, 2H, CO-CH_2-C**H**$_2$), 1.67 (q, J = 7.9, 7.5 Hz, 2H, N-CH_2-CH_2-C**H**$_2$), 1.27 (td, J = 7.1, 2.0 Hz, 3H, COO-CH_2-C**H**$_3$).

3.4.5 6-(12-methyl-7,14-dioxo-2,9-disulfo-12,14-dihydroquinolino[2,3-b]acridin-5(7H)-yl) hexanoic acid (Me−S_2quinacridone−COOH) (4)

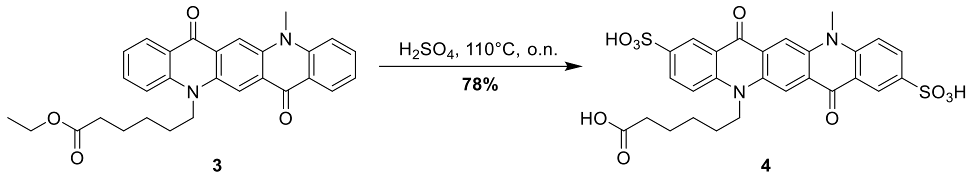

Me−Quinacridone−COOEt (**3**) (200 mg, 427 µmol, 1.0 equiv) was dissolved in conc. H_2SO_4 (5mL) and stirred at 110 °C overnight. The reaction was carefully poured on ice and neutralized with a saturated sodium hydroxide solution, followed by RP-MPLC (solvent A: water + 0.1% TFA, Solvent B: MeOH + 0.1% TFA, linear gradient from 20 - 50% B) to yield H−S_2quinacridone−COOH (**4**) as an orange powder (200mg, 333 µmol, 78%).

^{1}H NMR (400 MHz, MeOD): = 8.94 (d, J = 2.2 Hz, 1H, Ar5), 8.93 (d, J = 2.3 Hz, 1H, Ar1), 8.55 (s, 1H, Ar9), 8.50 (s, 1H, Ar10), 8.13 – 8.02 (m, 2H, Ar3, Ar7), 7.54 (pseudo-t, J = 8.5 Hz, 2H, Ar4, Ar8), 4.35 (t, J = 8.5 Hz, 2H, N-C**H**$_2$), 3.89 (s, 3H, Me), 2.42 (t, J = 7.3 Hz, 2H, CO-C**H**$_2$), 1.95 – 1.82 (m, 2H, N-CH$_2$-C**H**$_2$), 1.77 (p, J = 7.6 Hz, 2H, CO-CH$_2$-C**H**$_2$), 1.62 (p, J = 8.3 Hz, 2H, N-CH$_2$-CH$_2$-C**H**$_2$).

HR MS (ESI, neg. mode): m/z: calculated $C_{27}H_{25}N_2O_{10}S_2^{1-}$ $[M-H]^-$: 599.0789,
found: 599.0782 $[M-1H]^{1-}$, deviation: 1.2 ppm.

3.5 Synthesis of fluorescently labelled nucleotides

3.5.1 γ-O-(6-(S_2Acridone) amidohexyl) adenosine-O5'-triphosphate (5)

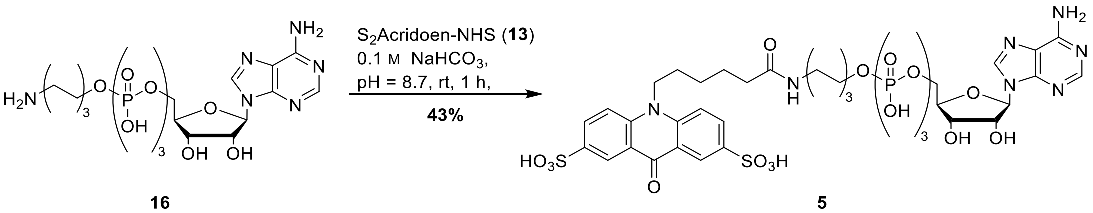

γ-O-(6-(S_2Acridone) amidohexyl) adenosine-O5'-triphosphate (**5**) was synthesized from γ-O-(6-aminohexyl) adenosine-O5'-triphosphate (**16**) (4.00 µmol, 1.0 equiv) according to the general procedure 2 (2 eq dye) and was obtained as a yellow powder (1.70 µmol, 43%).

^{1}H NMR (400 MHz, D_2O): δ = 8.82 (d, *J* = 2.3 Hz, 2H, Ar1, Ar8), 8.42 (s, 1H, H8), 8.24 (dd, *J* = 9.3, 2.3 Hz, 2H, Ar3, Ar6), 7.94 (s, 1H, H2), 7.91 (d, *J* = 9.3 Hz, 2H, Ar4, Ar5), 5.97 (d, *J* = 5.9 Hz, 1H, H-1'), 4.68 (t, *J* = 5.5 Hz, 1H, H-2'), 4.55 (t, *J* = 5.0, 4.5 Hz, 1H, H-3'), 4.46 (t, *J* = 8.5, 7.8 Hz, 2H, N-C**H**$_2$), 4.42 – 4.35 (m, 1H, H-4'), 4.33 – 4.23 (m, 2H, H-5'), 3.97 (q, *J* = 6.9 Hz, 2H, P-δ-O-C**H**$_2$), 3.14 (t, *J* = 6.8 Hz, 2H, CONH-C**H**$_2$), 2.29 (t, *J* = 7.1 Hz, 2H, CO-C**H**$_2$), 1.89 (p, *J* = 7.6 Hz, 2H, N-CH$_2$-C**H**$_2$), 1.71 (p, *J* = 7.3 Hz, 2H, CO-CH$_2$-C**H**$_2$), 1.61 (p, *J* = 6.8 Hz, 2H, P-δ-O-CH$_2$-C**H**$_2$), 1.58 – 1.50 (m, 2H, N-CH$_2$-CH$_2$-C**H**$_2$), 1.50 – 1.41 (m, 2H, CONH-CH$_2$-C**H**$_2$), 1.32 – 1.28 (m, 4H, linker).

^{31}P NMR (162 MHz, D_2O): δ = -10.83 (d, *J* = 19.3 Hz, 1P), -11.53 (d, *J* = 19.1 Hz, 1P), -23.21 (t, *J* = 19.1 Hz, 1P).

HR MS (ESI, neg. mode): m/z: calculated $C_{35}H_{44}N_7O_{21}P_3S_2^{2-}$ $[M-2H]^{2-}$: 527.5617,
found: 527.5642 $[M-2H]^{2-}$, deviation: 4.7 ppm.

3.5.2 δ-O-(6-(S_2Acridone) amidohexyl) adenosine-O5'-tetraphosphate (6)

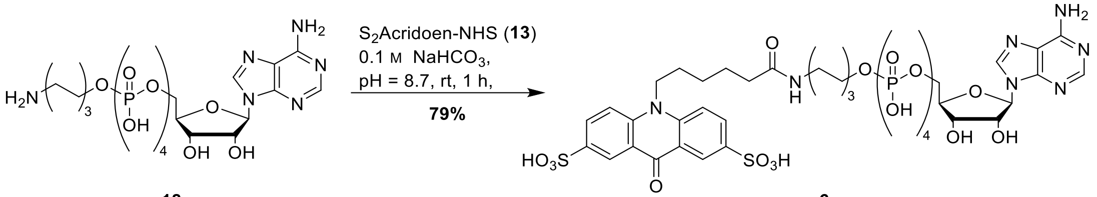

δ-O-(6-(S_2Acridone) amidohexyl) adenosine-O5'-tetraphosphate (**6**) was synthesized from δ-O-(6-aminohexyl) adenosine-O5'-tetraphosphate (**18**) (2.00 µmol, 1.0 equiv) according to the general procedure 2 (3.8 eq dye) and was obtained as a yellow powder (1.57 µmol, 79%).

^{1}H NMR (600 MHz, D_2O): δ = 8.84 – 8.81 (m, 2H, Ar1, Ar8), 8.46 (s, 1H, H8), 8.25 (dd, *J* = 9.2, 2.3 Hz, 2H, Ar3, Ar6), 7.94 (s, 1H, H2), 7.92 (d, *J* = 9.2 Hz, 2H, Ar4, Ar5), 5.98 (d, *J* = 6.2 Hz, 1H, H-1'), 4.73 (s, 1H, H-2'), 4.60 (t, *J* = 5.2, 4.1 Hz, 1H, H-3'), 4.46 (t, J = 8.1, 6.0 Hz, 2H, N-C**H$_2$**), 4.41 (bs, 1H, H-4'), 4.36 – 4.21 (m, 2H, H-5'), 4.01 (q, *J* = 6.8 Hz, 2H, P-δ-O-C**H$_2$**), 3.18 (t, *J* = 6.8 Hz, 2H, CONH-C**H$_2$**), 2.30 (t, *J* = 7.2 Hz, 2H, CO-C**H$_2$**), 1.88 (p, *J* = 8.4 Hz, 2H N-CH$_2$-C**H$_2$**), 1.71 (p, *J* = 7.8 Hz, 2H, CO-CH$_2$-C**H$_2$**), 1.66 (p, *J* = 6.8 Hz, 2H, P-δ-O-CH$_2$-C**H$_2$**), 1.57 – 1.46 (m, 4H N-CH$_2$-CH$_2$-C**H$_2$**, CONH-CH$_2$-C**H$_2$**), 1.43 – 1.33 (m, 4H, linker).

^{31}P NMR (243 MHz, D_2O): δ = -10.85 (d, *J* = 19.3 Hz, 1P), -11.26 – -11.83 (m, 1P), -22.84 – -23.95 (m, 2P).

HR MS (ESI, neg. mode): m/z: calculated $C_{35}H_{44}N_7O_{24}P_4S_2K^{2-}$ $[M\text{-}3H\text{+}K]^{2-}$: 586.5228,
found: 586.5226 $[M\text{-}3H\text{+}K]^{2-}$, deviation: 0.3 ppm.

3.5.3 ε-O-(6-(S_2Acridone) amidohexyl) adenosine-O5'-pentaphosphate (11)

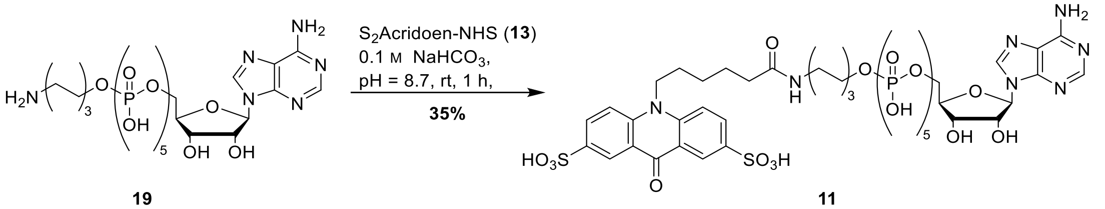

ε-O-(6-(S_2Acridone) amidohexyl) adenosine-O5'-pentaphosphate (**11**) was synthesized from ε-O-(6-aminohexyl) adenosine-5'-pentaphosphate (**19**) (2.00 µmol, 1.0 equiv) according to the general procedure 2 (4 eq dye) and was obtained as an orange powder (0.69 µmol, 35%).

^{1}H NMR (400 MHz, D_2O,): δ = 8.83 (d, *J* = 2.6 Hz, 2H, Ar1, Ar8), 8.51 (s, 1H, H8), 8.26 (d, *J* = 9.2 Hz, 2H, Ar3, Ar6), 7.99 (s, 1H, H2), 7.94 (d, *J* = 9.0 Hz, 2H, Ar4, Ar5), 6.03 (d, *J* = 6.7 Hz, 1H, H-1'), 4.77 (bs, 1H, H-2'), 4.64 (bs, 1H, H-3'), 4.48 (t, *J* = 7.7 Hz, 2H, N-C**H$_2$**), 4.44 (bs, 1H, H-4'), 4.39 – 4.18 (m, 2H, H-5'), 4.03 (q, *J* = 6.7 Hz, 2H, P-δ-O-C**H$_2$**), 3.19 (t, *J* = 6.6 Hz, 2H, CONH-C**H$_2$**), 2.31 (t, *J* = 6.3 Hz, 2H, CO-C**H$_2$**), 1.98 – 1.80 (m, 2H, N-CH$_2$-C**H$_2$**), 1.76 – 1.63 (m, 4H, CO-CH$_2$-C**H$_2$**, P-δ-O-CH$_2$-C**H$_2$**), 1.62 – 1.48 (m, 4H, N-CH$_2$-CH$_2$-C**H$_2$**, CONH-CH$_2$-C**H$_2$**), 1.47 – 1.33 (m, 4H, linker).

^{31}P NMR (162 MHz, D_2O): δ = -10.78 (d, J = 16.1 Hz, 1P), -11.48 (d, J = 16.6 Hz, 1P), -22.40 – -24.47 (m, 3P).

HR MS (ESI, neg. mode): m/z: calculated $C_{35}H_{45}N_7O_{27}P_5S_2K^{3-}$ $[M\text{-}4H\text{+}K]^{3-}$: 417.3345,
found: 417.3346 $[M\text{-}4H\text{+}K]^{3-}$, deviation: 0.2 ppm.

4 NMR Spectra of all compounds

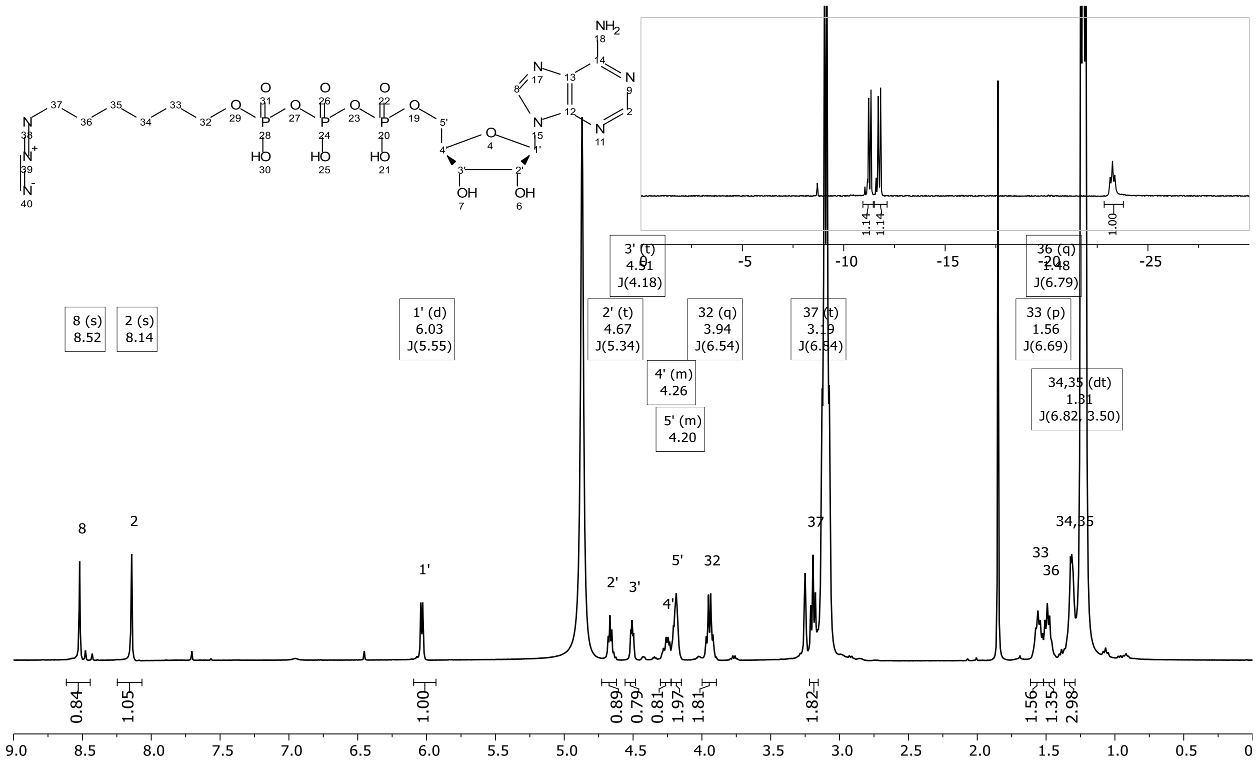

^{1}H NMR (400 MHz, D_2O): γ-O-(6-Azidohexyl) adenosine-O5'-triphosphate (**15**). Inlet: -^{31}P NMR (162 MHz, D_2O).

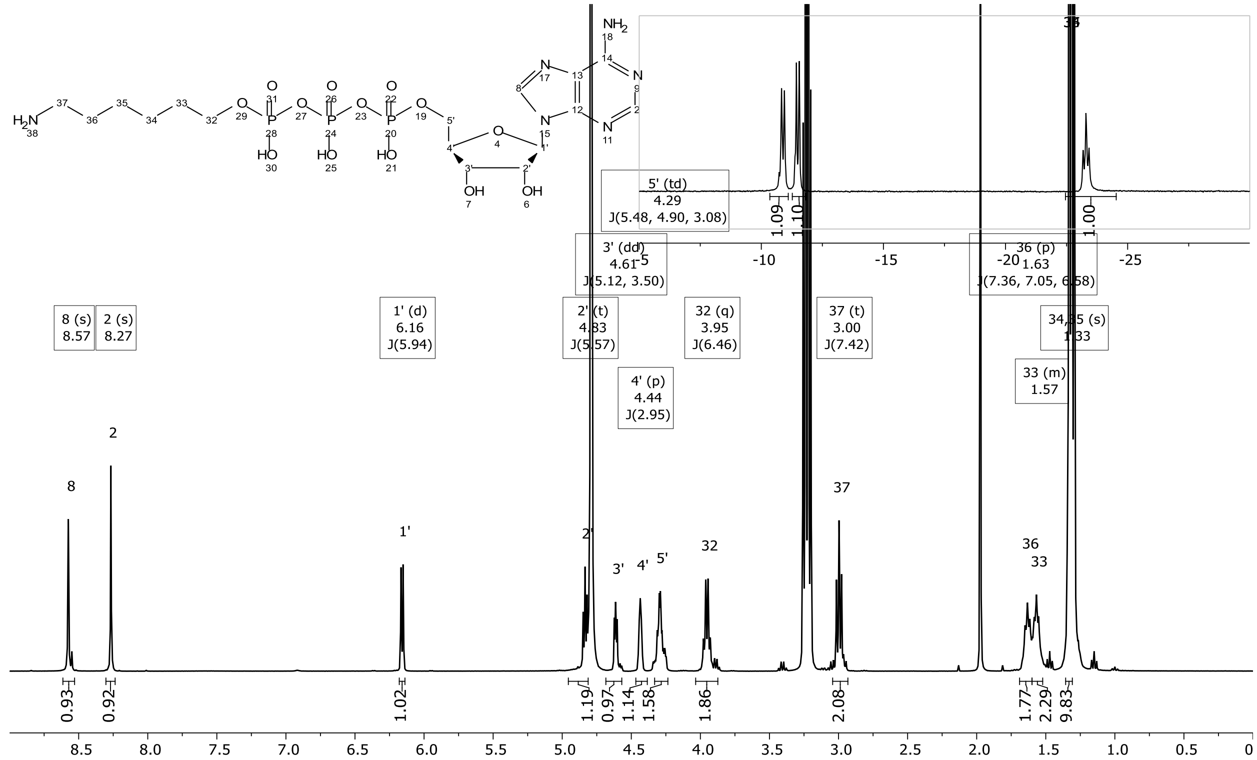

^{1}H NMR (400 MHz, D_2O): γ-O-(6-Aminohexyl) adenosine-O5'-triphosphate (**16**).
Inlet: ^{31}P NMR (162 MHz, D_2O).

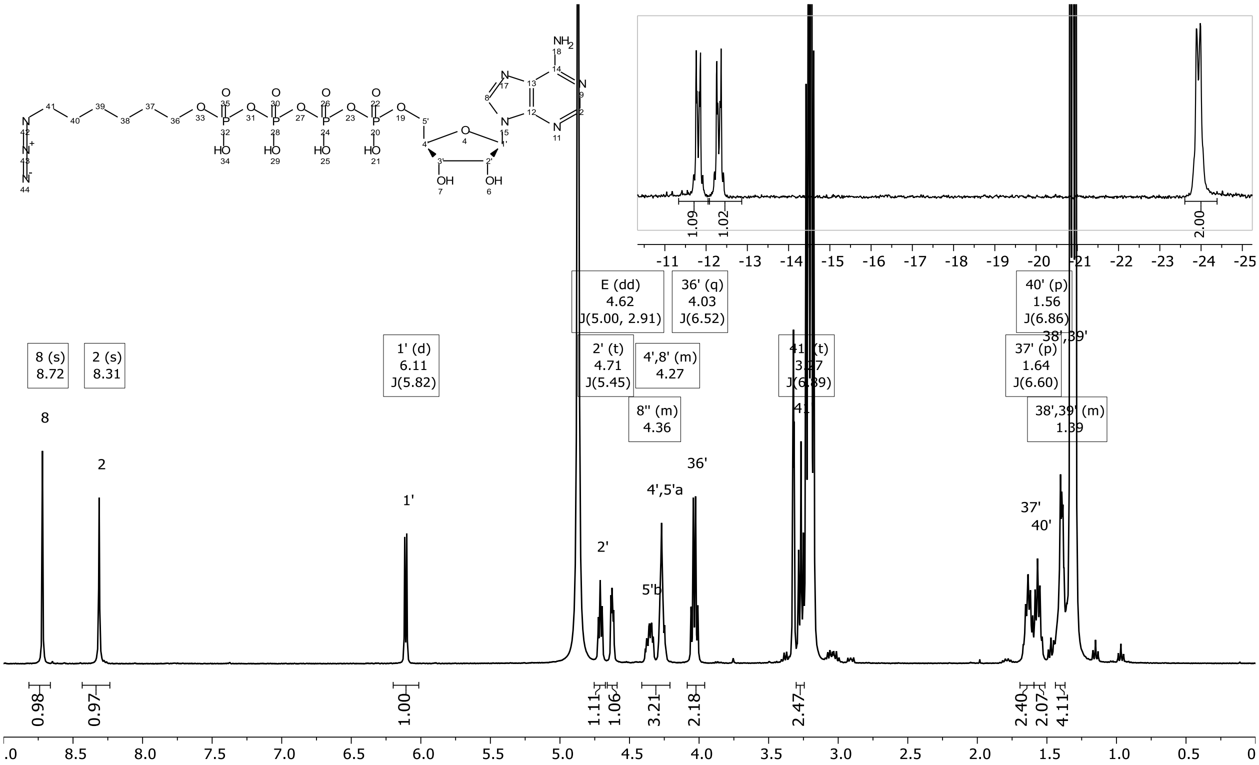

^{1}H NMR (400 MHz, MeOD): δ-(6-Azidohexyl)-adenosine-O5′-tetraphosphate (**17**).

Inlet: ^{31}P NMR (162 MHz, D_2O).

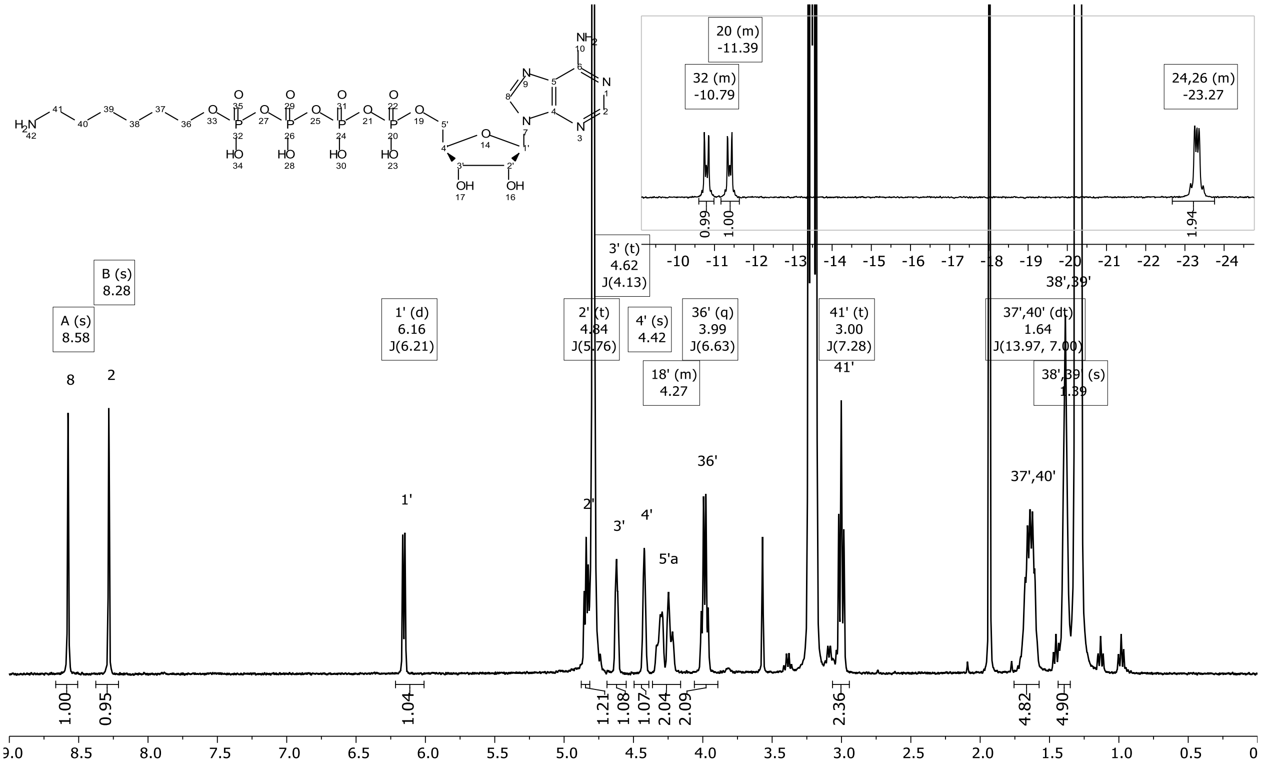

^{1}H NMR (400 MHz, D_2O): γ-O-(6-Aminohexyl) adenosine-O5′-tetraphosphate (**18**).

Inlet: ^{31}P NMR (162 MHz, D_2O).

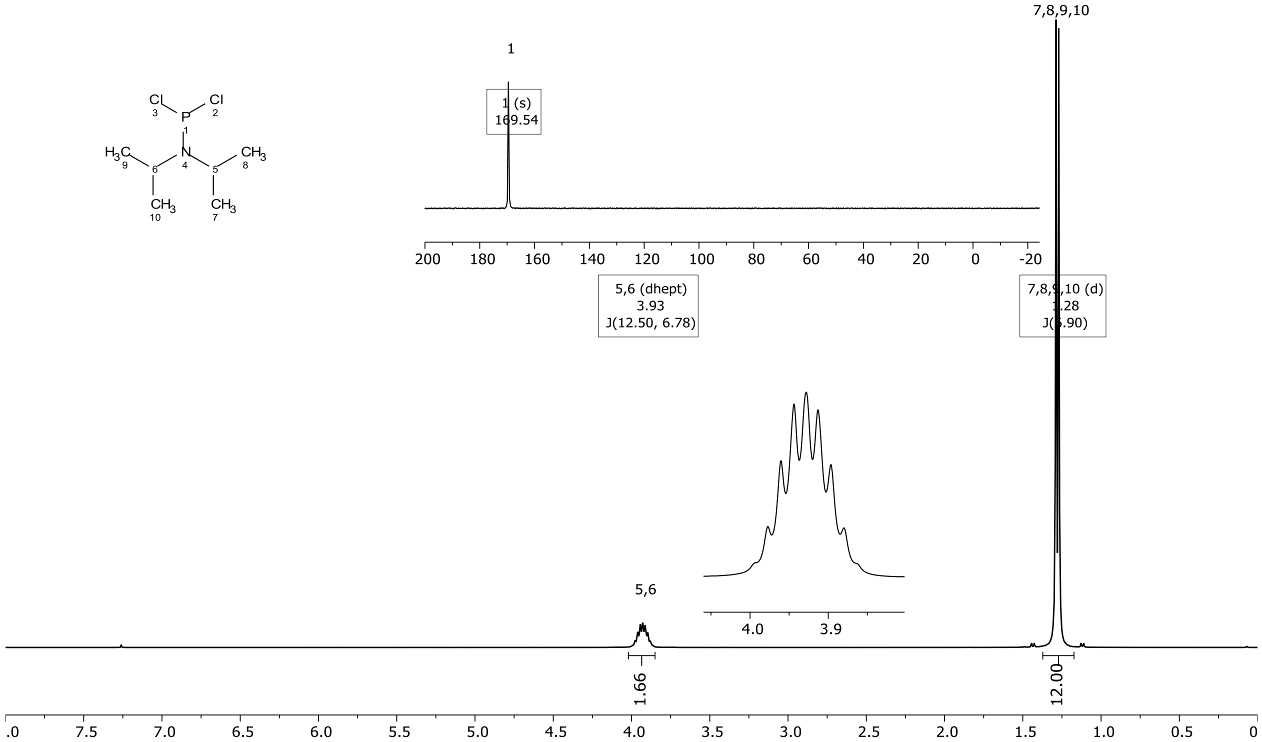

^{1}H NMR (400 MHz, $CDCl_3$): Diisopropylamino dichlorophosphine (**7**).

Inlet: ^{31}P NMR (162 MHz, $CDCl_3$).

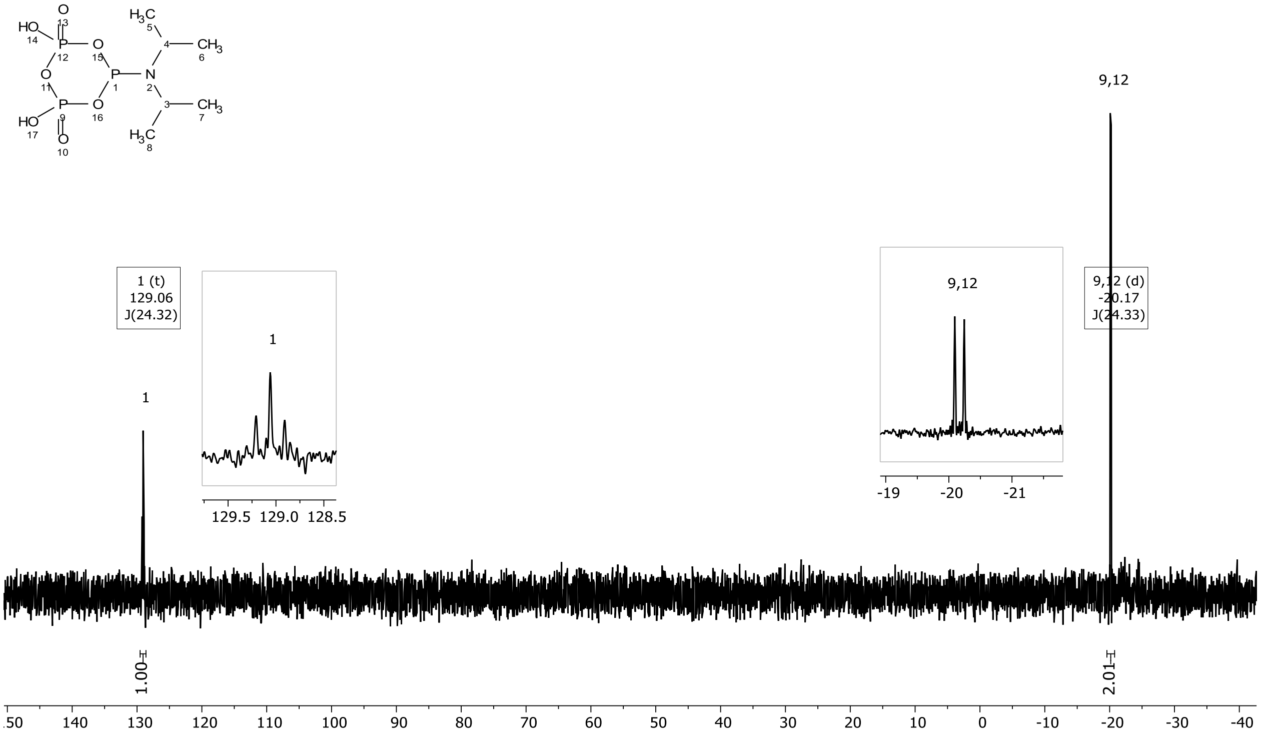

^{31}P NMR (162 MHz, DMF-d7): Cyclic pyrophosphoryl diisopropylphosphoramidite (**8**).

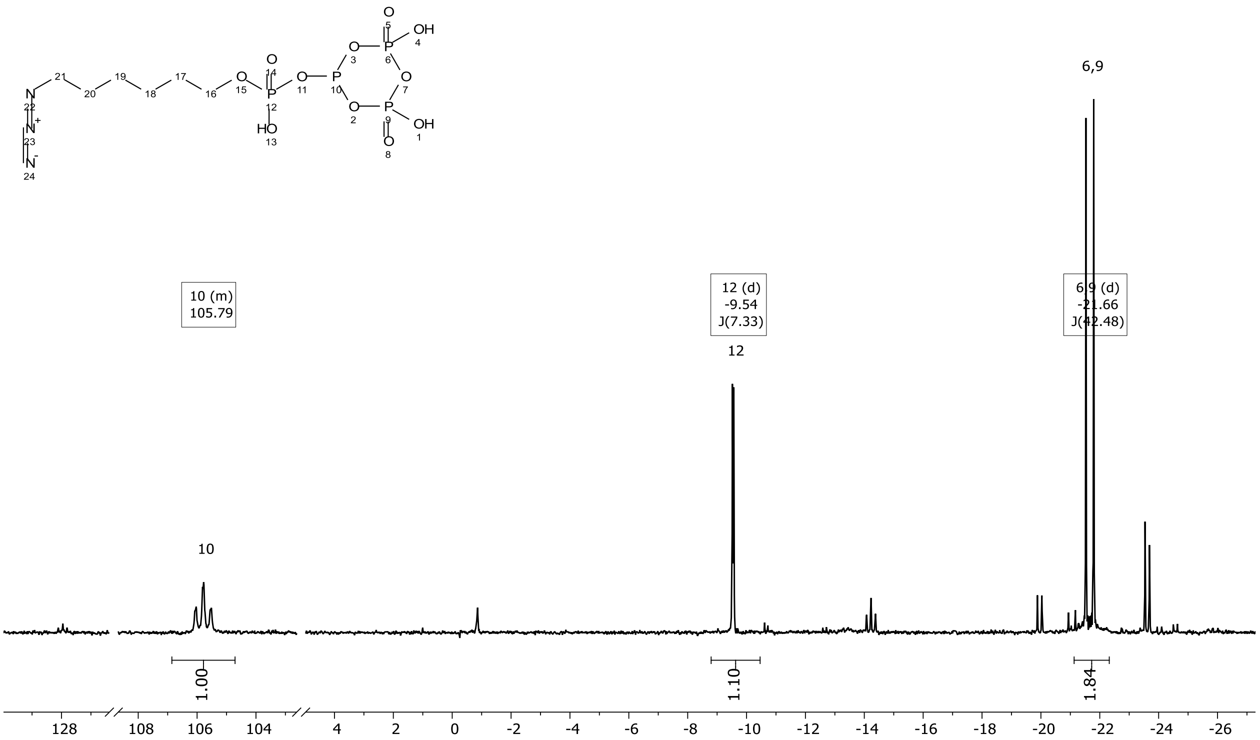

^{31}P NMR (162 MHz, DMF-d7): 1-(6-Azido)hexyl phosphoryl cyclotriphosphate (unoxidized **9_red**).

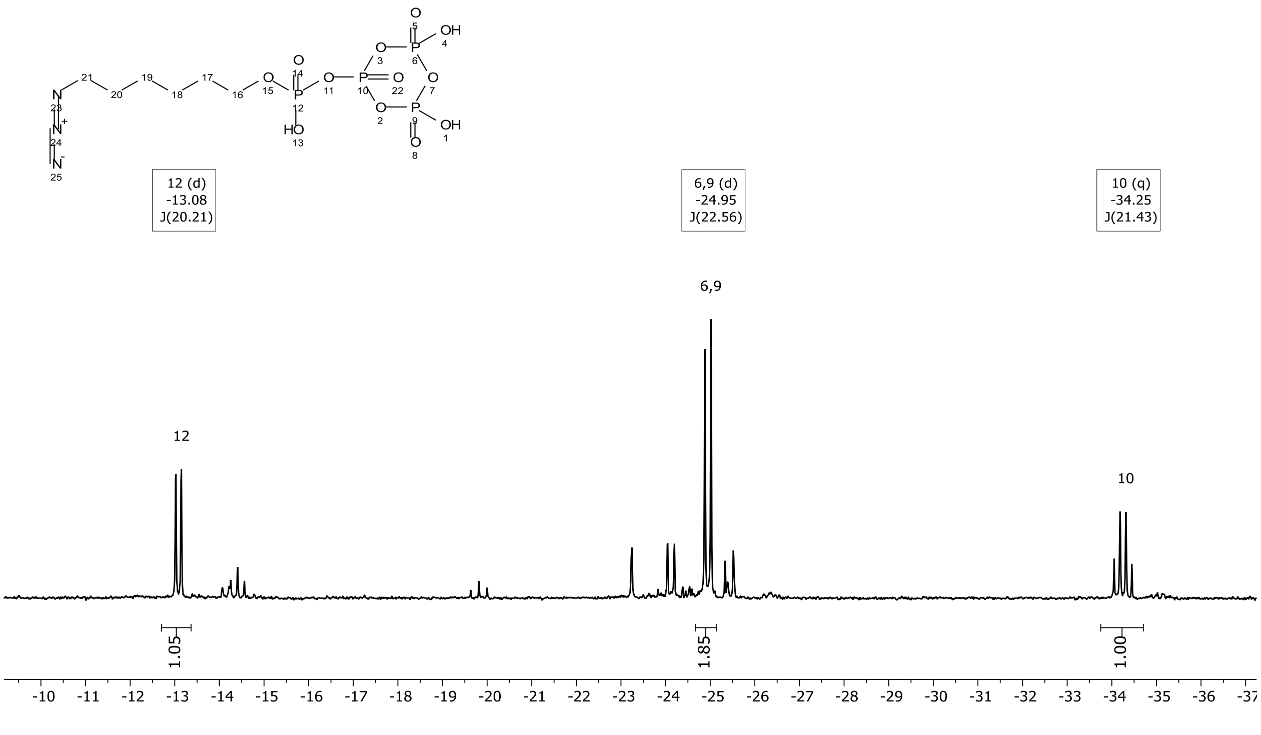

^{31}P NMR (162 MHz, DMF-d7): 1-(6-Azido)hexyl phosphoryl cyclotriphosphate (**9**).

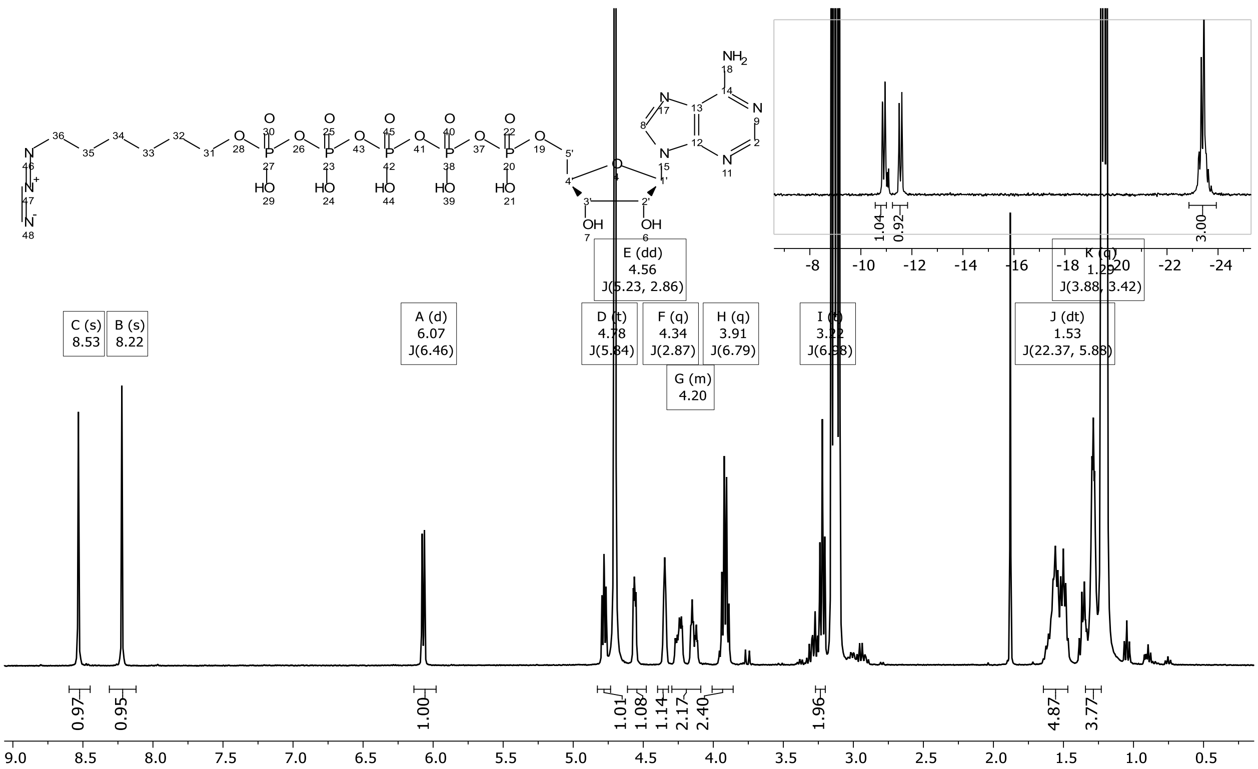

^{1}H NMR (400 MHz, D_2O): ε-(6-Azidohexyl)-adenosine-O5'-pentaphosphate (**10**).
Inlet: ^{31}P NMR (162 MHz, D_2O).

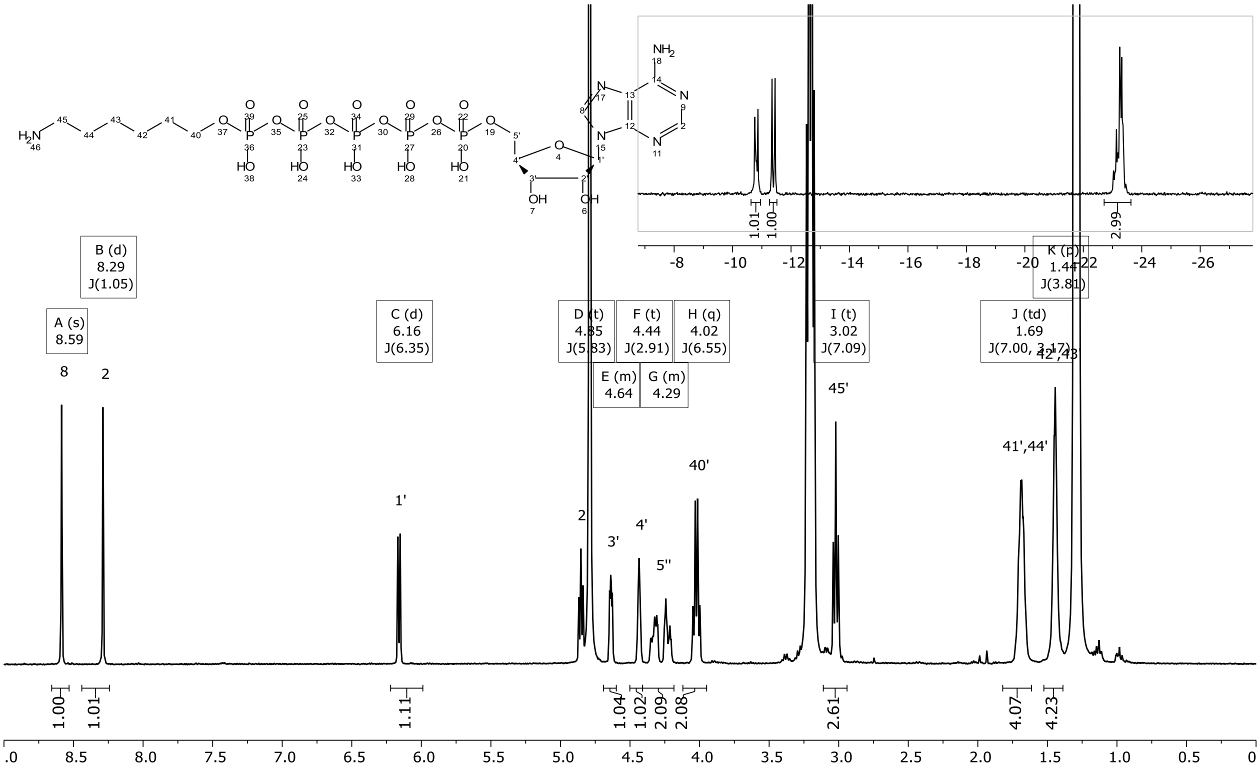

^{1}H NMR (400 MHz, D_2O): ε-(6-Aminohexyl)-adenosine-O5'-pentaphosphate (**19**).
Inlet: ^{31}P NMR (162 MHz, D_2O).

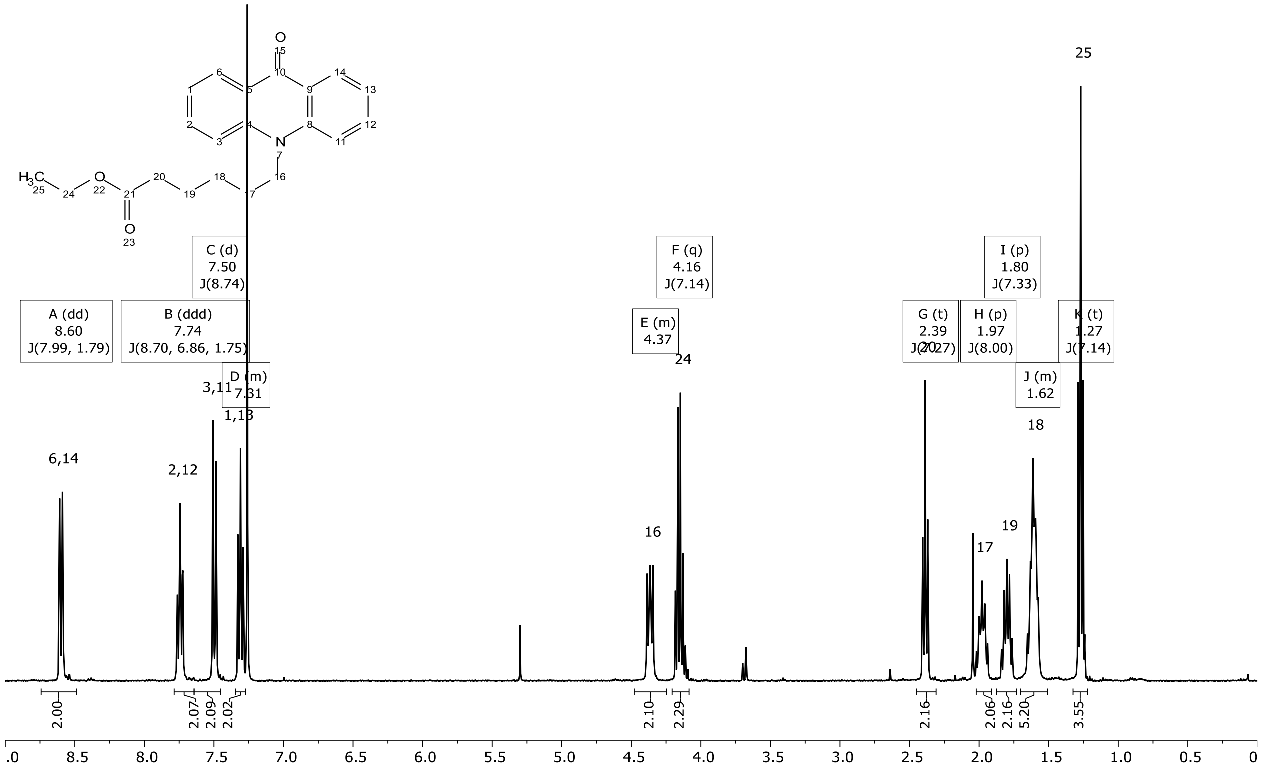

^{1}H NMR (400 MHz, $CDCl_3$): Ethyl 6-(9-oxo-9H-acridin-10-yl)-hexanoate (**1**).

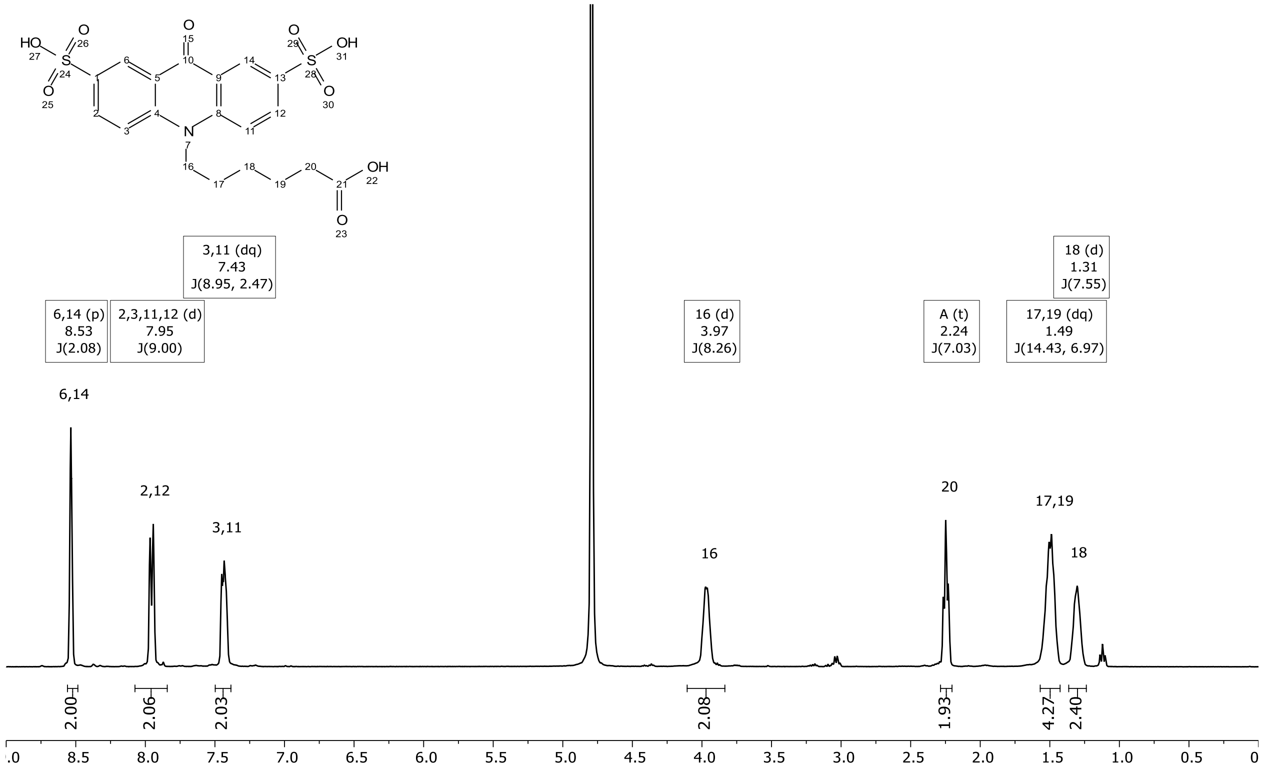

^{1}H NMR (400 MHz, D_2O): Ethyl 6-(9-oxo-9H-acridin-10-yl)-hexanoate 6-(9-oxo-2,7-disulfoacridin-10(9H)-yl)hexanoic acid (**2.2**).

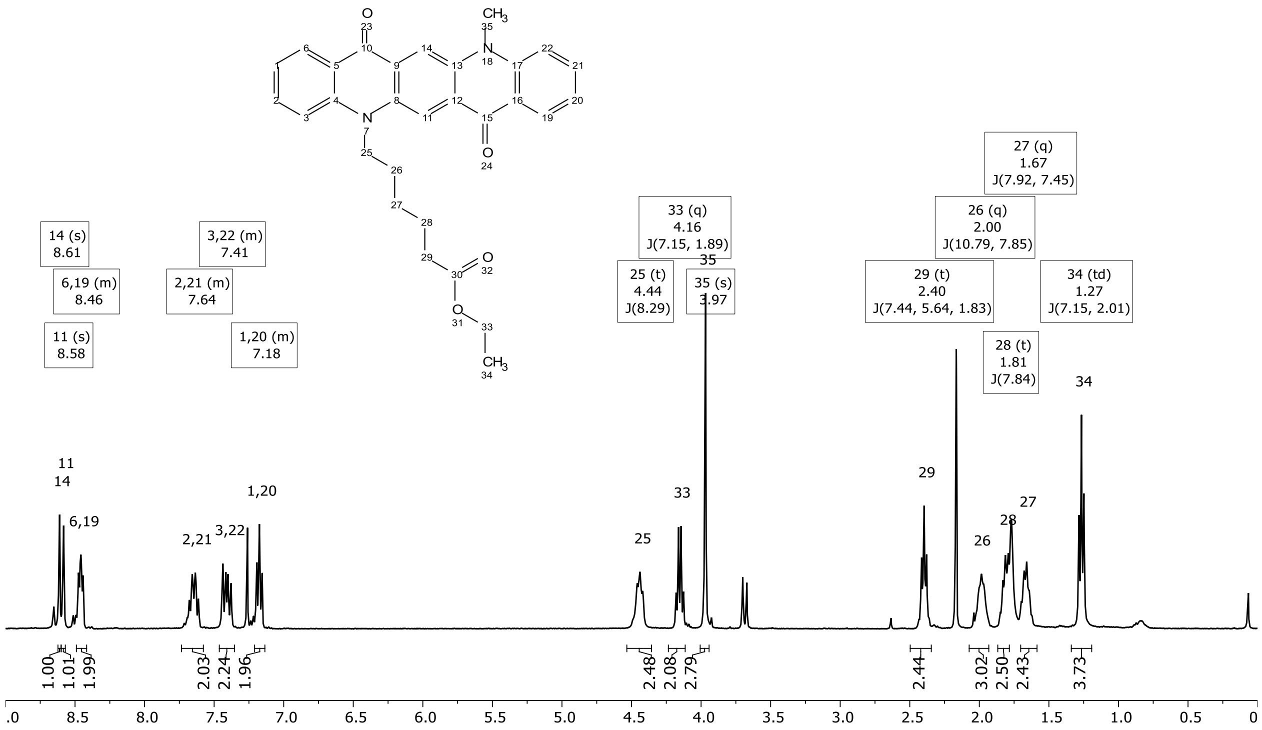

^{1}H NMR (400 MHz, $CDCl_3$): Ethyl 6-(12-methyl-7,14-dioxo-12,14-dihydroquinolino[2,3-b]acridin-5(7H)-yl)hexanoate (Me-quinacridone-COOEt) (**3**).

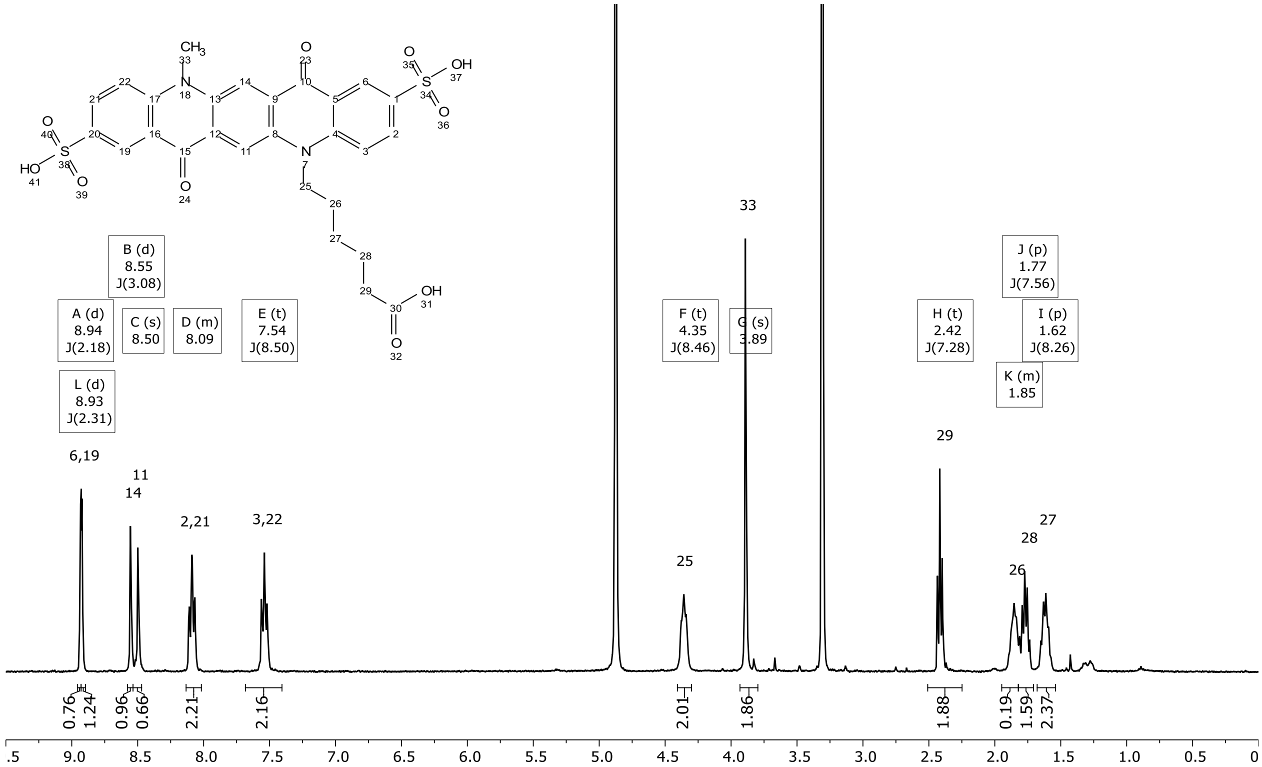

^{1}H NMR (400 MHz, MeOD): 6-(12-methyl-7,14-dioxo-2,9-disulfo-12,14-dihydroquinolino[2,3-b]acridin-5(7H)-yl) hexanoic acid (Me−S_2quinacridone−COOH) (**4**).

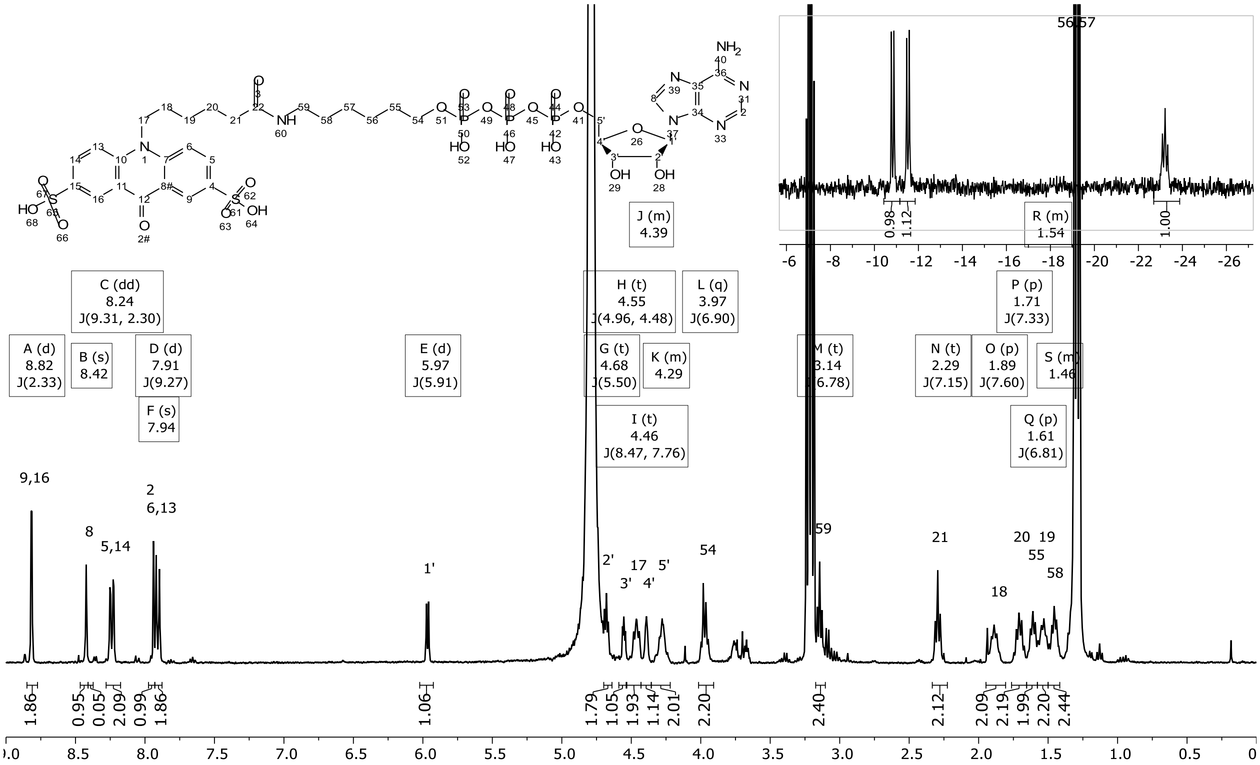

^{1}H NMR (400 MHz, D_2O, 2 amide rotamers): γ-O-(6-(S_2Acridone) amidohexyl) adenosine-O5'-triphosphate (**5**)

Inlet: ^{31}P NMR (162 MHz, D_2O).

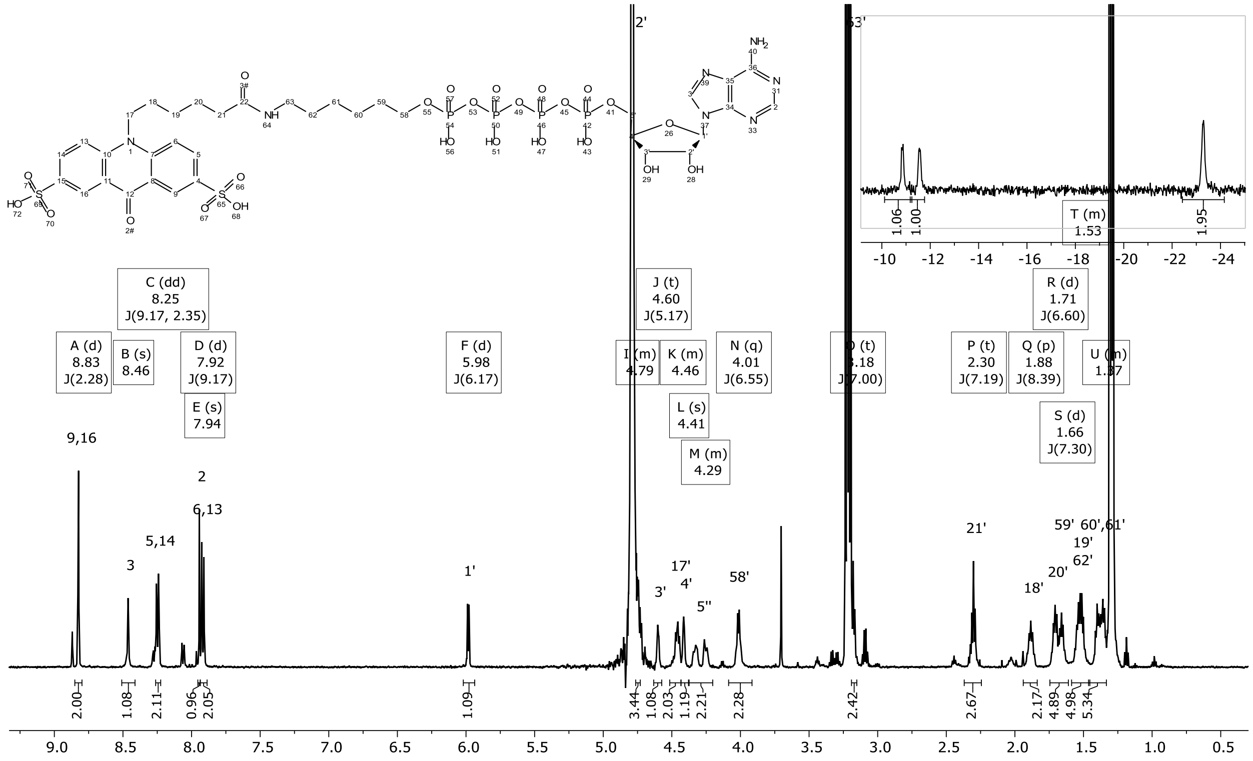

^{1}H NMR (600 MHz, D_2O): δ-O-(6-(S_2Acridone) amidohexyl) adenosine-O5'-tetraphosphate (**6**)

Inlet: ^{31}P NMR (243 MHz, D_2O).

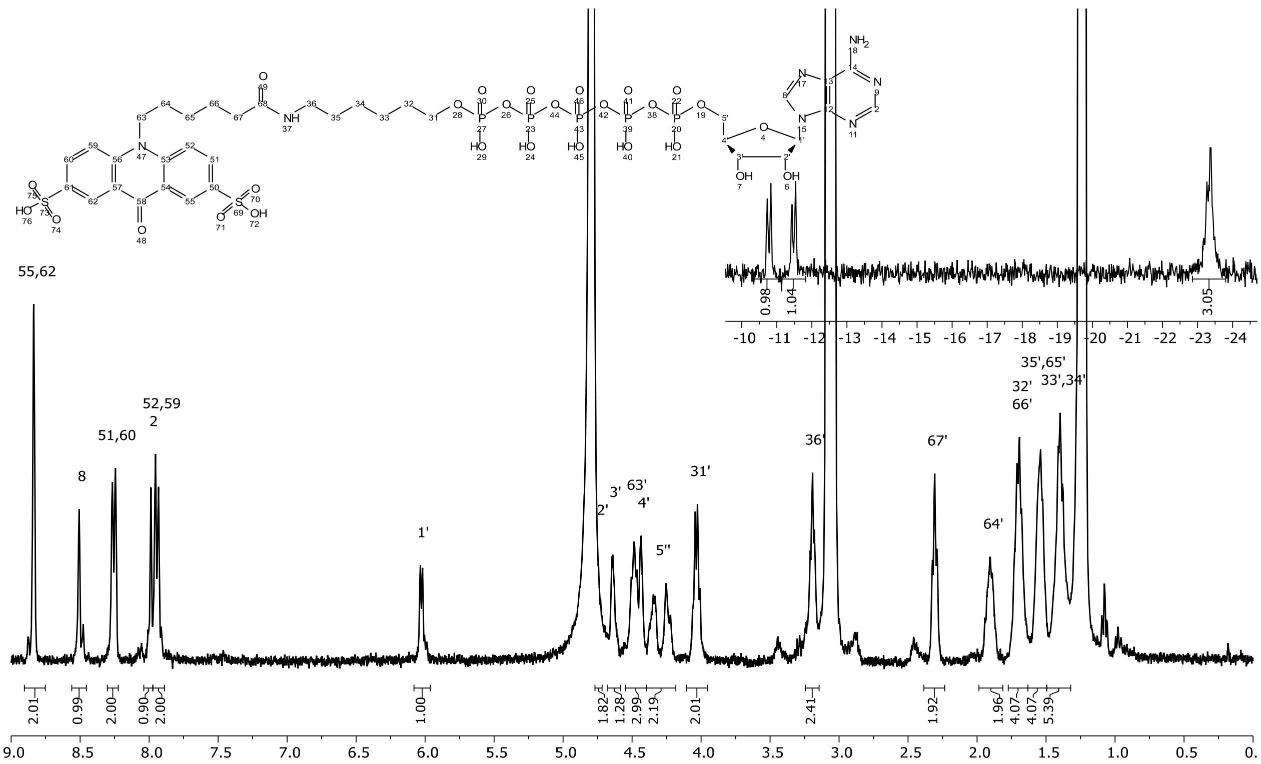

^{1}H NMR (400 MHz, D_2O): ε-O-(6-(S_2Acridone) amidohexyl) adenosine-O5'-pentaphosphate (**11**)
Inlet: ^{31}P NMR (162 MHz, D_2O).

5 Absorption/emission spectra

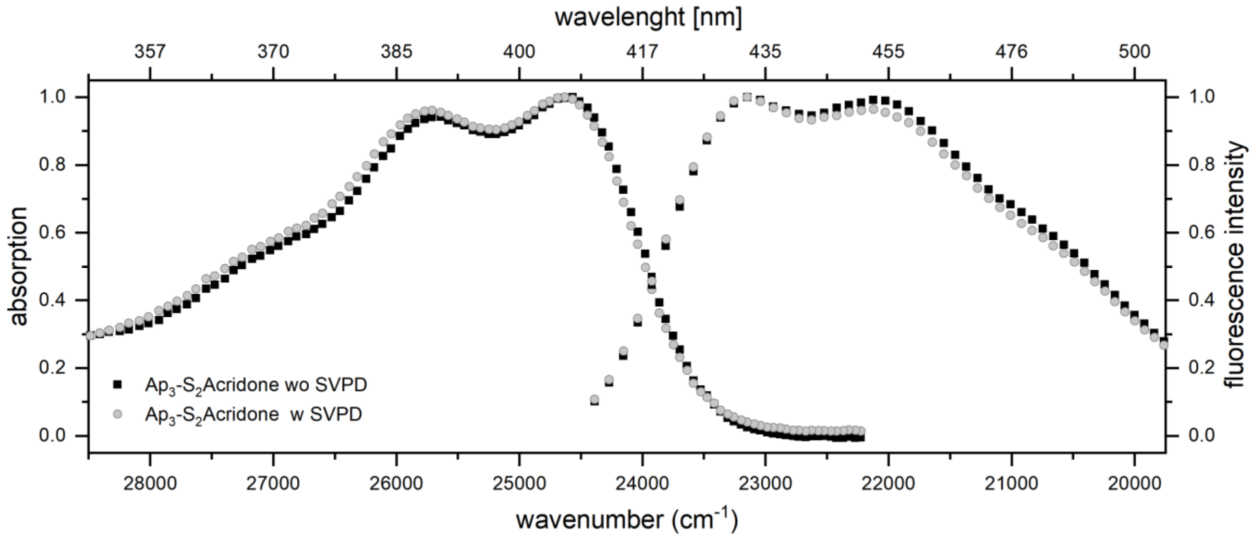

γ-O-(6-(S_2Acridone) amidohexyl) adenosine-O5'-triphosphate (**5**).

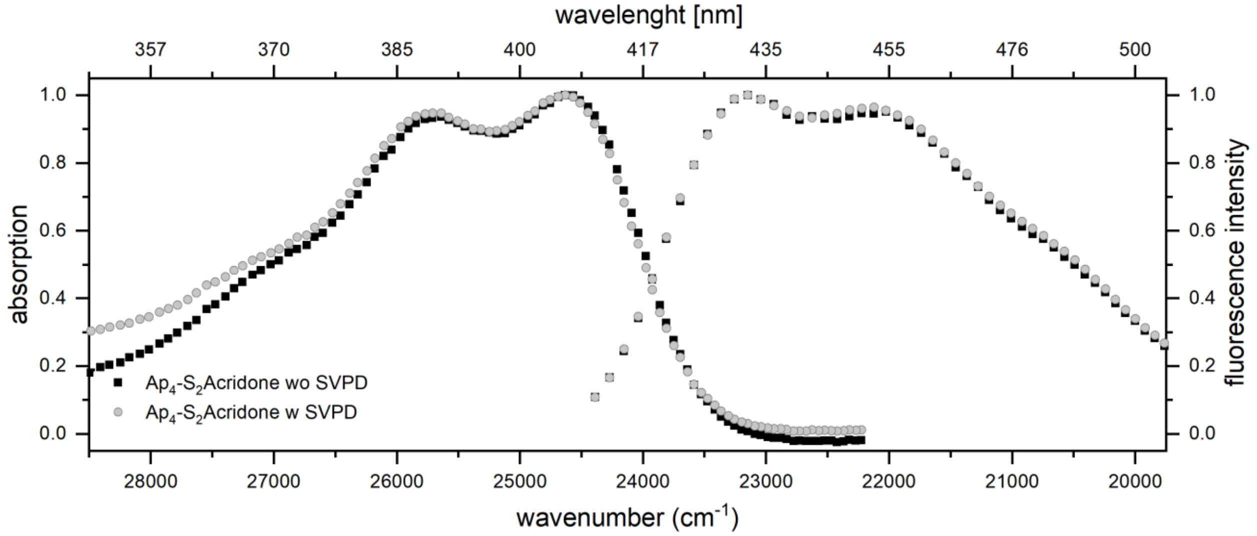

δ-O-(6-(S_2Acridone) amidohexyl) adenosine-O5'-tetraphosphate (**6**).

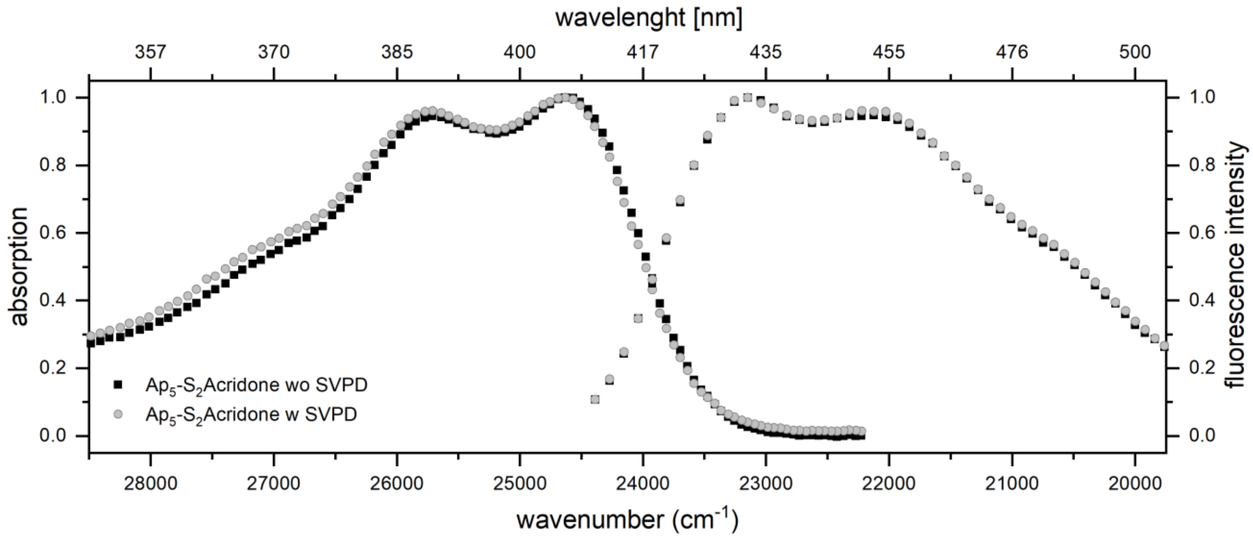

ε-O-(6-(S_2Acridone) amidohexyl) adenosine-O5'-pentaphosphate (**11**).

<u>Supplementary References:</u>

1. C. E. Berndsen and C. Wolberger, *Anal. Biochem.*, **2011**, 418, 102-110.
2. K. H. Götz, M. Mex, K. Stuber, F. Offensperger, M. Scheffner and A. Marx, *Cell Chemical Biology*, **2019**, 26, 1535-1543.e1535.
3. F. Mortensen, D. Schneider, T. Barbic, A. Sladewska-Marquardt, S. Kuhnle, A. Marx and M. Scheffner, *Proc. Natl. Acad. Sci. U. S. A.*, **2015**, 112, 9872-9877.
4. C. Sailer, F. Offensperger, A. Julier, K. M. Kammer, R. Walker-Gray, M. G. Gold, M. Scheffner and F. Stengel, *Nature communications*, **2018**, 9, 4441.
5. S. E. Lee, L. M. Elphick, A. A. Anderson, L. Bonnac, E. S. Child, D. J. Mann and V. Gouverneur, *Bioorg. Med. Chem. Lett.*, **2009**, 19, 3804-3807.
6. C. Romuald, E. Busseron and F. Coutrot, *The Journal of Organic Chemistry*, **2010**, 75, 6516-6531.
7. S. M. Hacker, M. Mex and A. Marx, *The Journal of Organic Chemistry*, **2012**, 77, 10450-10454.
8. M. Mex, *AG Prof. Andreas Marx*, **2011**, Bachelor Thesis.
9. F. Song, J. Zhang, Y. Zhao, W. Chen, L. Li and Z. Xi, *Org. Biomol. Chem.*, **2012**, 10, 3642-3654.
10. J. Singh, N. Steck, D. De, A. Hofer, A. Ripp, I. Captain, M. Keller, P. A. Wender, R. Bhandari and H. J. Jessen, *Angew. Chem. Int. Ed*, **2019**, 58, 3928-3933.
11. J. A. Smith, R. M. West and M. Allen, *Journal of Fluorescence*, **2004**, 14, 151-171.
